# The Role of Breast Cancer Stem Cells as a Prognostic Marker and a Target to Improve the Efficacy of Breast Cancer Therapy

**DOI:** 10.3390/cancers11071021

**Published:** 2019-07-20

**Authors:** Maria Giovanna Scioli, Gabriele Storti, Federico D’Amico, Pietro Gentile, Giulia Fabbri, Valerio Cervelli, Augusto Orlandi

**Affiliations:** 1 Anatomic Pathology Institute, Department of Biomedicine and Prevention, University of Rome Tor Vergata, 00133 Roma, Italy; 2 Plastic and Reconstructive Surgery, Department of Surgical Sciences, University of Rome Tor Vergata, 00133 Roma, Italy

**Keywords:** breast cancer stem cells, epithelial–mesenchymal transition, circulating tumor cells, metastasis, therapy resistance, breast cancer stem cell-targeted strategies

## Abstract

Breast cancer is the most common form of tumor in women and the leading cause of cancer-related mortality. Even though the major cellular burden in breast cancer is constituted by the so-called bulk tumor cells, another cell subpopulation named cancer stem cells (CSCs) has been identified. The latter have stem features, a self-renewal capacity, and the ability to regenerate the bulk tumor cells. CSCs have been described in several cancer types but breast cancer stem cells (BCSCs) were among the first to be identified and characterized. Therefore, many efforts have been put into the phenotypic characterization of BCSCs and the study of their potential as prognostic indicators and therapeutic targets. Many dysregulated pathways in BCSCs are involved in the epithelial–mesenchymal transition (EMT) and are found up-regulated in circulating tumor cells (CTCs), another important cancer cell subpopulation, that shed into the vasculature and disseminate along the body to give metastases. Conventional therapies fail at eliminating BCSCs because of their quiescent state that gives them therapy resistance. Based on this evidence, preclinical studies and clinical trials have tried to establish novel therapeutic regimens aiming to eradicate BCSCs. Markers useful for BCSC identification could also be possible therapeutic methods against BCSCs. New approaches in drug delivery combined with gene targeting, immunomodulatory, and cell-based therapies could be promising tools for developing effective CSC-targeted drugs against breast cancer.

## 1. Introduction

Breast cancer is the highest incidence cancer and the leading cause of cancer-related mortality among women [1]. It cannot be considered a single disease because genetic and genomic variability together with clinicopathological features determine different stages and prognoses [2]. Even though in breast cancer the major cellular burden is constituted by the so-called bulk tumor cells, other cell subpopulations with stem features, self-renewal capacity, and the ability to regenerate the bulk tumor cells can be identified [3]. Because of similarities with the stem progenitors of normal tissues, these cells have been defined cancer stem cells (CSCs). They have been described in several cancers but breast cancer stem cells (BCSCs) were among the first to be identified and characterized [4].

The first evidence of their role in tumorigenesis were inferred from the injection into a xenograft mouse model. CSCs were able to recreate the tumor while other cellular subtypes from the tumor bulk were not, hence they have also been defined as tumor-initiating cells [4]. It remains to be clarified whether these cells derive from a stem cell that has undergone malignant transformation, or are the result of stem-program activation and dedifferentiation in a tumor cell [5].

BCSCs have also been extensively studied for years because of two main features that are crucial in cancer prognosis and progression: (1) their capacity to induce the epithelial–mesenchymal transition (EMT), to undergo self-renovation, and ultimately to give birth to new bulk tumor cells [6]; and (2) their resistance to conventional therapies [7]. Many efforts have been put into a better characterization and identification of BCSCs in order to verify their prognostic value and their usefulness in the monitoring of therapeutic efficacy. CD44 and CD24 were among the first studied markers in order to identify the CSC population [4]. Later, the enzyme aldehyde dehydrogenase (ALDH or ALDH-1) was identified as another marker of CSCs [8]. Combined analysis for ALDH-1, CD44, and CD24 demonstrated the existence of two populations that partially overlapped but were not identical. However, they were both able to recreate a tumor in a xenograft, thus suggesting the existence of several sub-populations of CSCs [8]. In parallel, a dysregulation of pathways of stemness and self-renewal such, as Wnt [9], PI3K/Akt/FOXO [10], TGF-β [11], and Notch [12], was found in CSCs; the same pathways were involved in tumor invasiveness, hematogenous spreading, and ultimately in metastases. Moreover, CSCs have been identified among the main actors in these processes [13,14]. Many of the pathways identified are involved in the EMT, a cardinal step in cancer diffusion [15]. This is a process of trans-differentiation from epithelial cells to mesenchymal cells, which are able to enter systemic circulation and diffuse to distant sites [16]. Genes like *SNAIL*, *SLUG* and *TWIST* are overexpressed in cells undergoing EMT [17], in CSCs [18] and in circulating tumor cells (CTCs) [19]. CSCs are capable to acquire both an epithelial/proliferating and a mesenchymal/invasive phenotype [20]. They demonstrate a great plasticity and the capacity to switch between these two phenotypes playing probably a crucial role in EMT [21]. Different CSC subpopulations have been identified among the pool of CTCs, confirming their capacity to enter the blood stream and spread distantly [19]. Therefore, the enumeration of CTCs and the identification of the circulating CSCs among CTCs have been proposed as possible prognostic factors, as well as indicators of disease progression and metastatic risk [22]. Therapies based on traditional clinicopathological markers, that usually target the tumor bulk, fail in eliminating CSCs [7]. The quiescent state of CSCs inside the tumor microenvironment allows them to resist conventional drugs, which target mainly proliferating cells [23]. Then, the CSCs’ ability to proliferate and regenerate the tumor burden ultimately leads to relapse or progression of the disease [7]. Preclinical studies and clinical trials have tried to establish novel therapeutic regimens that aim to eradicate also the stem component in the tumor for a complete control of the disease [24,25,26]. In order to have a holistic approach to the tumor system, new and conventional drugs have been combined together in order to address bulk and BCSCs at the same time [27].

Many useful markers for the characterization and identification of CSCs can be both possible therapeutic targets to eliminate BCSCs and indicators of response to therapy. Among these markers, there are molecules involved mainly in self-renewal and survival, such as Notch, Hedgehog, Wnt, PI3K/Akt/mTOR, IL-8, HER2 and the TGF-β pathway [27]. New technologies in drug delivery, combined with gene targeting, differentiating agents, immunomodulatory, and cell-based therapies, are promising tools for developing effective CSC-targeted drugs against breast cancer.

## 2. Breast Cancer Stem Cells as Markers for Prognosis and Therapy Monitoring

### 2.1. Breast Cancer Stem Cells and Circulating Tumor Cells (CTCs)

As reported above, the epithelial–mesenchymal transition (EMT) is a crucial step in disease progression. EMT is an embryonic program that is re-activated in tumor cells. It confers features proper of mesenchymal cells to epithelial, which are non-motile cells, and gives them the ability to invade adjacent tissues and to disseminate under the influence of multiple cytokines, which are produced by the surrounding stroma [28]. CSCs represent one of the leading actors in this process, which includes their transformation into circulating tumor cells (CTCs) [15]. Given this close link to metastasis, CTCs have been studied for several years as a possible marker of metastatic disease (Table 1) [29] and they have been correlated to a worse prognosis in metastatic breast cancer [30]. In 2004, the first prospective multicentric study, on metastatic breast cancer patients, demonstrated that five CTCs per 7.5 mL of peripheral blood was the best cut-off value in order to identify patients with a worse prognosis, and a reduced overall survival (OS) and progression-free survival (PFS).

In 2014, another multicentric study, undertaken on 1944 patients, confirmed the threshold of 5 CTCs per 7.5 mL as the most effective in order to stratify metastatic breast-cancer patients with worse prognosis and to create a better predictive model [31]. In this study, the baseline CTC-count was an independent prognostic factor for OS and PFS. The baseline CTC-count was able to improve the prognostication of OS and PFS when it was added to a full clinicopathological model. A further improvement in the predictive ability of this model was attained through the addition of the CTC count at 3–5 weeks and at 6–8 weeks.

CTCs can be detected in peripheral blood even in the initial stages of the disease. A pooled analysis of data from 3173 patients with non-metastatic breast cancer (Stage I to III) demonstrated the presence of one or more CTCs in 20.2% of the patients [32]. In this study, one CTC, or more, could be used as an independent prognostic factor for OS and disease-free survival (DFS).

Several ongoing clinical trials have measured CTCs in order to orient therapeutic decisions (DETECT III (NCT01619111); CirCe T-DM1 (NCT01975142); Treat CTC trial (NCT01548677; [33,34,35]). Many of these trials have not presented the final survival analysis yet but results from published data are still conflicting.

Even though all the evidence confirms that CTCs have a high prognostic value, a lot of open issues still deserve further debate in order to be clarified.

All the studies cited above used the CellSearch^TM^ (Veridex) system, which is the only one approved by the Food and Drug Administration for the CTC detection in peripheral blood [36] (CELLSEARCH^®^ Circulating Tumor Cell Kit (Epithelial) Instructions for Use. Janssen Diagnostics, LLC). It is a system based on the epithelial cell adhesion molecule (EpCAM), which targets CTCs with an epithelial phenotype only, thus having several limitations. For instance, circulating CSCs play an active role in the progression of the disease in metastases and in drug resistance. With the CellSearch^TM^ system, either they are cut-out from the count, or they are counted together with purely epithelial CTCs.

Standardized detection systems have not dealt with the extreme heterogeneity of CTCs yet. Furthermore, the significance of the CTC phenotype analysis still remains to be clarified.

In both EpCAM-positive and EpCAM-negative CTCs, the expression of Notch1, an important stemness marker, was correlated with brain metastases [12,37]. In an ongoing trial, Notch1 has been used as a CTC marker (together with HER2, COX-2, EGFR, and ST6GALNAC5), in order to evaluate, through the CTC count, the risk of brain recurrences after focal radiotherapy (NCT02941536).

The estrogen receptor/progesterone receptor (ER/PR) status of CTCs was compared to that of the primary tumor in several studies. A concordance in between 40% and 70% was reported [38,39,40,41]. Clinical significance of this discrepancy and the possible impact on therapeutic choices have not been evaluated yet.

Similarly, the HER2/neu status on CTCs matched only in a limited number of cases with that of the primary tumor. In two recent studies, the concordance rate of HER2/neu between CTCs and the primary tumor was about 60% [41]. The clinical significance of this discrepancy is yet to be understood. In a metanalysis by Wang et al. [42], the CTCs’ HER2/neu status was a prognostic factor in non-metastatic patients only. One or more HER2/neu-positive CTCs were associated with a reduced OS and PFS. Clinical trials are ongoing in order to verify whether the treatment with drugs against HER2/neu could be beneficial in patients with HER2/neu positive CTCs, independently from the HER2/neu status of the primary tumor (NCT01619111 and NCT01975142).

Similarly, the genotypic analysis of CTCs for mutations of estrogen receptor gene *ESR1* [43] for the altered HER2 expression [41], for mutations in the PIK3CA gene [44], and for the expression of stemness markers, as with the ALDH-1 gene [45], is gaining ever more attention.

The different cellular populations among CTCs, which recapitulate those present in the primary tumor, are another critical point that should be considered. It is possible to distinguish epithelial CTCs, mesenchymal CTCs, and CTCs with a staminal phenotype, which could be both epithelial-mesenchymal (EM) or mesenchymal-epithelial (ME) [19,46,47]. In this sense, a merely quantitative evaluation of the CTCs is not able to distinguish the prognostic meaning of the different CTC subpopulations.

CTC analysis can give a better insight into CSCs and their dynamic evolution during a cancer’s natural history. The identification of stemness and EMT markers on CTCs could possibly help us in order to create an in depth profile of the primary tumor and the metastases and to target more precisely those cell populations, which are mostly responsible of the resistance to therapies, of the disease dissemination, and ultimately of a worse prognosis [4,24,48]. For instance, 41% of patients with aggressive breast cancers (triple negative or HER2 + tumors) presented mesenchymal CTCs, which are not measured by the standard systems [19].

AKT2, PI3Kα, and twist-related protein 1 (TWIST1) are the three main markers that are expressed by the epithelial-mesenchymal (EM) CTCs. These markers were evaluated by several groups in order to identify EM-CTCs [24,49,50]. Patients with nodal involvement and metastatic breast cancer had a higher presence in systemic circulation of CTCs with the EM phenotype [51,52]. An observational study analyzed epithelial CTCs, EM CTCs, and purely mesenchymal CTCs on 56 metastatic breast-cancer patients [53]. Both epithelial CTCs and EM CTCs were significantly associated with a poorer OS, while only EM CTCs were correlated with a reduced PFS.

Another multicentric prospective study used a microfluidic system for the detection of different CTC populations and tried to establish a threshold in order to predict the PFS after one year [54]. The patients with a total number of CTCs equal or superior to 10 and with a proportion of mesenchymal CTCs greater than 10.7% had a worse median PFS compared to the patients who did not meet these criteria.

Nonetheless, the methods used to count the different CTC populations are yet to be standardized and validated, and their prognostic and predictive value is unclear.

Among CTCs, the main markers associated with circulating CSCs are ALDH-1, CD44, and CD24, which are able to identify the cells with a higher metastatic potential [17,55,56]. The breast CSCs can switch easily in between the EM phenotype (which is EpCAM^−^CD49f^+^ and expresses the CSC markers CD44^+^/CD24^−^) and the ME phenotype (which is EpCAM^+^CD49f^+^ and expresses the CSC marker ALDH-1^+^) [20]. Circulating CSCs with the EM phenotype can be identified with EpCAM-based systems, but it is not possible to distinguish them from the purely epithelial CTCs without further analysis [6]. Matrigel invasion assays with these two circulating CSC subpopulations (EM-CSCs and ME-CSCs) showed that CSCs with an EM phenotype have a greater invasive capacity than ME CSCs [20]. This could be in favor of the theory that hypothesizes that CSCs from the primary tumor undergo EMT on the invasive front and enter the circulation, thus spreading to distant sites. Micro-metastases are quiescent until these CSCs revert to a mesenchymal/epithelial, “self-renewing” phenotype, and originate the new bulk tumor. Nonetheless, the clinical impact of these markers expressed on CTCs remains unclear.

The analysis and the dosage of circulating tumor DNAs and micro-RNAs are other promising options in order to profile CSCs and to evaluate the minimal residual disease [57]. A Taiwanese study dosed the levels of two micro-RNA (miR-9 and miR-221) in 206 patients. MiR-9 and miR-221 have been associated with stemness features, elevated metastatic potential, and EMT activation. In this study, high levels of MiR-9 and miR-221 were independently associated with a poorer OS and DFS after 8 years of follow-up [58]. Nonetheless, the prognostic and predictive values of these markers are still far to be fully understood [59].

### 2.2. Analysis of the Cancer Stem Cells in the Primitive Tumor

Even though it is easier to access and to monitor blood parameters like stem CTCs over time, the analysis of the tumoral specimens still remains of pivotal importance in order to characterize CSCs and try to understand disease prognosis and the possible response to therapies. ALDH-1, CD44, and CD24 were among the first markers that were evaluated regarding primary tumors through immunohistochemistry in order to identify the CSC population, even though the data about their clinical prognostic value are still contradictory. Quantitative immunofluorescence for ALDH-1^+^ and CD44^+^/CD24^−^ cells was retrospectively evaluated on 639 patients with 12.6 years of follow up. Co-expression of these markers correlated significantly with a worse outcome independently of the tumor size, grade, nodal status, and HER2/neu and receptor status, while the ALDH-1 alone did not significantly predict an outcome [60]. In a study on 144 patients with invasive ductal carcinoma, neither immunohistochemical ALDH-1^+^ nor CD44^+^/CD24^−^ correlated with a difference in OS [61]. In another study on 121 patients, a positive immunohistochemical staining, both for ALDH and CD44/CD24, was evaluated. A positive staining for ALDH-1 was significantly correlated with a higher rate of metastasis or recurrence [62]. The staining for CD44^+^/CD24^−^ cells was not significantly associated per se with metastasis or recurrence even though a higher proportion of these cells in the tumor showed a significant association with metastatic disease and recurrences.

Other authors highlighted that ALDH-1 is a marker of invasiveness and metastatic potential, while the CD44^+^/CD24^−^ ratio indicates mainly a “self-renewal” capacity, thus these two markers are assigned different functions during the tumor progression. Therefore, they advocated always combining the use of both the markers for the sake of a better understanding of the stem population [63].

Many authors tried to develop a molecular “fingerprint” of the tumor in order to have a deeper comprehension of CSC role in breast cancer evolution and to draw reliable prognostic conclusions. Gwak et al. analyzed the tumoral expression of several transcription factors proper of the embryonic stem cells, including Oct4, Sox2, Nanog, Bmi1, and Klf4 [64]. Expression of Oct4 correlated with ALDH-1 positivity, a high Ki-67 and a high histological grade, and it was an independent prognostic factor for a reduced DFS. These associations were found specifically in the hormone receptor (HR)-positive group and in the HR-positive patients in treatment with tamoxifen. This is possibly another hint in favor of a previously suggested association between Oct-4 and tamoxifen resistance [65]. It was proposed that one should combine different multi-gene prognostic signatures that address 17 specific genes (HTICS), each of them with roles in three different major pathways in the tumor biology: immune response, cell migration, and cell proliferation [66]. These genes were selected among those expressed more by the tumor-initiating cells than by the non-tumor-initiating ones, thus obtaining a prognostic tool that was able to predict metastasis-free survival (MFS) and OS in HER2^+^/ERα^−^ cancer patients. Despite the reduction of the signature to a six-gene panel, the authors highlighted that there was still a significant prognostic value, even if reduced.

The importance of the selection of a genetic signature of CSCs and not only of the bulk tumor has been recently demonstrated and clinically validated for a panel of 20 genes in order to add prognostic and predictive value to clinical models. [67]. This 20-gene signature was selected among a set of stem-cell-specific genes overexpressed in mammary stem cells. The genetic signatures derived from bulk tumoral cells frequently overlapped clinicopathological features, thus reducing their prognostic ability in cases like triple-negative breast cancers, which lack expression of hormone receptors and have a high proliferation rate. This panel was clinically validated on a cohort of 2453 breast cancers and it was able to predict the risk of distant metastases in triple-negative (TNBC) and luminal breast cancers, independent of standard clinicopathological parameters.

A better understanding of CSCs both circulating and quiescent in the primitive tumor bulk can lead to a better prognosis prediction, therapy allocation, and ultimately to the development of targeted treatments for these cells, which are often resistant to conventional chemotherapy.

## 3. Breast Cancer Stem Cell-Targeting: New Strategies in Drug Development for Therapy Resistance

**Figure 1:**
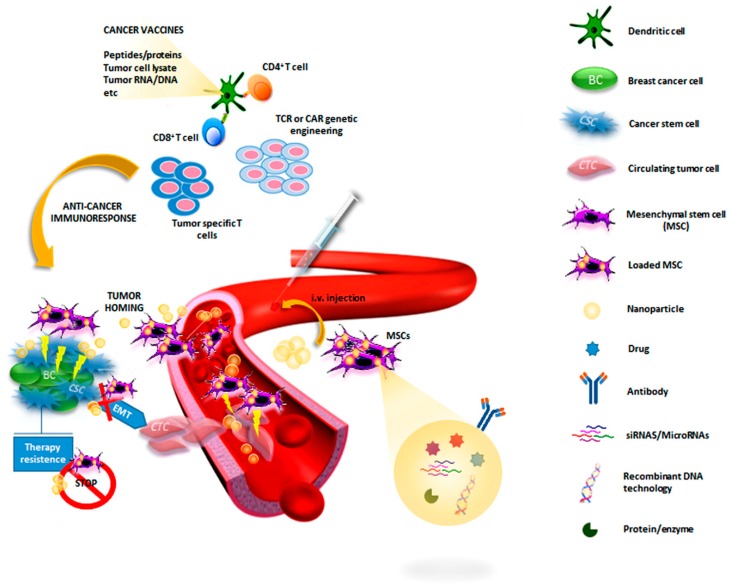
An overview of the main strategies based on BCSC-targeting. After systemic application, engineered immune cells or mesenchymal stem cells (MSCs), tailored to the molecular profile of patients’ breast cancer, home in on the tumor microenvironment and release different specific anti-CSC drugs (proteins, enzymes, recombinant DNA, miRNAs, siRNAs, and chemotherapeutics). Nanoparticles containing different anti-CSC molecules can be administered alone or incorporated into MSCs to reach the tumor microenvironment and deliver drugs.

Conventional drugs targeting the tumor bulk are ineffective at eradicating CSCs [7]. In particular, it has been reported that anti-mitotic agents, such as taxanes (paclitaxel and docetaxel), cannot target quiescent CSCs inside the tumor bulk [23], leading to the reconstitution of the initial tumor cell population, increasing the adhesiveness of CTCs and the disease progression [68,69]. Based on this evidence, some studies have focused on directly targeting CSC subpopulation with promising results from preclinical experiments and clinical trials [24,25,26]. Novel therapeutic strategies for BCSC-targeting are based on the combined use of new and conventional drugs [27]. In particular, the main targets for BCSCs are Notch, Hedgehog, Wnt, PI3K/Akt/mTOR, IL-8, HER2, and TGF-β signaling, which are implicated in BCSC self-renewal and survival [27]. Moreover, the application of nano and biotechnologies, combined with gene targeting, represents a promising strategy for the development of effective BCSC-targeted drugs. An overview of the main strategies based on BCSC-targeting is shown in Figure 1.

### 3.1. Signaling Pathways Activated in Breast Cancer Stem Cells

#### 3.1.1. Notch Signaling

The most clinically developed approach is the inhibition of the Notch signaling by γ-secretase inhibitors (GSIs). Notch receptors are cleaved by γ-secretase, which determines the release of the Notch intracellular domain (NCID), and subsequently, Notch signaling activation. Then, NCID is translocated to the nucleus where it induces gene transcription by interacting with other co-factors [70,71]. Notch signaling is highly active in BCSCs and it associates with tumor invasiveness [72]. The use of γ-secretase inhibitors has been proven effective in blocking the Notch pathway [72] and BCSC capability to form mammospheres in vitro [73]. Different phase I/II clinical trials using the γ-secretase inhibitor MK-0752 (Merck) in combination with docetaxel are ongoing for the treatment of metastatic breast cancer [73]. Enrolled patients’ biopsies demonstrated a significant reduction in BCSC number, sustaining the advantages of Notch pathway inhibitors for BCSC-targeted therapy. In addition, other GSIs are in use to treat metastatic breast cancer such as RO4929097 (also combined with paclitaxel and carboplatin), PF-03084014, LY3039478, and CB-103 (Table 2).

#### 3.1.2. Hedgehog Signaling

Hedgehog signaling is implicated in the maintenance of CSC stemness and in the regulation of self-renewal, survival, angiogenesis, EMT, and cell invasion [74]. Different inhibitors of the Hedgehog pathway, such as vismodegib, have been investigated, also in clinical trials, for their anti-CSC activity. In particular, an ongoing phase I clinical trial is evaluating the effects of vismodegib and RO4929097 on BCSC differentiation markers (Table 2). Sims-Mourtada et al. co-treated breast cancer cells with vismodegib and docetaxel and found a decrease in the BCSC number and mammosphere formation that were increased instead by docetaxel alone [75]. In addition, they reported a Hedgehog signaling-dependent induction of multi-drug resistance 1 (MDR1) and ATP-binding cassette super-family G member 2 (ABCG2) in BCSCs. The Hedgehog inhibitor vismodegib (GDC-0449) has also been proven to counteract tumor growth in tamoxifen-resistant breast cancer xenografts [76].

#### 3.1.3. Wnt Pathway

It has been reported that the Wnt pathway is highly active in BCSCs compared with the remaining tumor cells [77]. Different targets for Wnt pathway inhibition have been investigated, such as Porcupine O-Acyltransferase (PORCN), R-spondin-3 (RSPO3), Wnt family member 2B (WNT2B), Frizzled-5 (FZD5), FZD10, Tyrosine-protein kinase transmembrane receptor 1 (ROR1), tankyrase, and β-catenin, and some drugs have reached clinical trials [78] (Table 2). In particular, monoclonal antibodies targeting the Wnt pathway, such as vantictumab (OMP-18R5) and cirmtuzumab (UC-961), anti-Frizzled and anti-ROR1, have proven effective, in combination with paclitaxel, in treating metastatic breast cancer. A clinical trial is recruiting breast cancer patients in order to determine the effective dose of LGK-974 (WNT974), a PORCN inhibitor, that counteracts the palmitoylation and secretion of Wnt ligands, alone or in combination with immunotherapy (an anti-Programmed Death 1/PD-1 antibody). LGK-974 has proven effective in different in vitro and in vivo cancer models [79]. Moreover, two clinical trials are using Foxy-5, a Wnt5a mimicking peptide, as an anti-metastatic cancer drug. In a study by Hallett et al., the Wnt signaling inhibitor PKF118-310 has been reported to be effective in reducing tumor growth and BCSC number in HER2-overexpressing breast cancer xenografts [80]. ROR1 is a type I orphan receptor expressed exclusively on different tumor cell types [81]. ROR1 is mainly involved in EMT and metastasis and its silencing in breast cancer cells counteracted these processes in vivo [81].

#### 3.1.4. PI3K/Akt/mTOR Pathway

The PI3K/AKT/mTOR pathway regulates BCSC functions [52]. The inhibition of the PI3K/Akt/mTOR pathway by everolimus (RAD001) has been reported to counteract BCSC proliferation in primary breast cancer cells, especially in combination with docetaxel [114]. Combined treatment of everolimus and an aromatase inhibitor increased the progression-free survival in advanced hormone receptor-positive breast cancer patients [93] (Table 2).

#### 3.1.5. Ephrin/Ephrin Receptor Pathway

Ephrin receptors, belonging to the largest family of receptor tyrosine kinases (RTKs), have been reported to influence BCSC activity [115] and several molecules targeting this pathway are being tested in clinical trials, especially the tyrosine kinase inhibitors. Ephrin A4 has been reported to be a potential therapeutic target for BCSCs [116,117]. An antibody-drug conjugate targeting Ephrin A4, named PF-06647263, consisting of a humanized monoclonal antibody anti-Ephrin A4 conjugated to the DNA-damaging agent calicheamicin, has been developed and tested to induce tumor regression in TNBC xenografts in vivo [117]. Moreover, PF-06647263 is currently being evaluated in a phase I clinical trial in metastatic TNBC patients (Table 2).

#### 3.1.6. Chemokine Ligand 8-Chemokine Receptor Type 1/2 (CXCL8-CXCR1/2) Axis

Many studies have focused attention on the role of IL-8 (CXCL8) in the biology of BCSCs. CXCL8 is a chemokine whose biological effects are mediated by two G-protein-coupled receptors: CXCR1 and CXCR2 [118]. CXCL8 has been reported to play multiple roles in cancer, such as increasing proliferation, angiogenesis, and metastases [119], as well as in mammosphere formation in HER2-positive breast cancer [120]. The adding of exogenous CXCL8 has proven to be effective in promoting the proliferation of CSCs in vitro; this growth was prevented by the presence of CXCR1/2 antagonists, such as reparixin [121] or a monoclonal antibody anti-CXCR1 (but not anti-CXCR2) [122]. These results have also been confirmed in breast cancer cell lines and in breast cancer patient-derived xenografts, in which the combination of docetaxel and reparixin was more effective in reducing tumor growth than either treatment alone, with a significant decrease in CSC number through apoptosis by activating Fas/FasL signaling [122] (Table 2). In addition, the combined treatment with reparixin and paclitaxel in the human TNBC cell line MDA-MB231 showed a synergistic effect, as proven for mammosphere activity and cell cycle arrest, likely mediated by the inhibition of focal adhesion kinase (FAK)/AKT and cyclin B1 signaling [123]. Neutralizing anti-CXCR1 and anti-CXCL8 monoclonal antibodies induced the same results [123]. SCH563705, another CXCR1/2 inhibitor, has been reported to counteract the effects of exogenous CXCL8 on BCSC mammosphere activity [120]. Currently, two clinical trials (phase I and II) are evaluating the efficacy of reparixin on BCSC survival (the first in combination with paclitaxel) (Table 2). In the ongoing phase Ib clinical study [124], patients with HER-2 negative metastatic breast cancer who received reparixin and paclitaxel showed no cytotoxic effects and are in long-term remission [124]. However, it was not possible to collect serial biopsies of tumor tissue at baseline and during treatment. No significant differences in CTC number, ALDH expression, and EMT transcription factors were observed, likely due to the small sample size and high baseline heterogeneity.

A pilot trial (NCT01861054) of single agent reparixin that is evaluating the efficacy of reparixin to eliminate CSC in primary operable breast cancer [97] found the same limitations. A randomized, placebo-controlled clinical trial is using paclitaxel with and without reparixin in a front-line treatment of metastatic TNBC with good tolerability [124].

#### 3.1.7. EGFR/HER2 and TGF-β Signaling

Several molecules targeting EGFR/HER2 signaling (downstream of IL-8) have been intensively investigated in breast cancer. In particular, Lapatinib (a HER2 inhibitor) has proven effective in counteracting tumor growth of HER2-positive and negative breast cancers, showing moderate toxicity and a decrease in brain metastases [125]. Currently, some clinical trials are evaluating the efficacy of Lapatinib on BCSCs [25] (Table 2). Trastuzumab (Herceptin), an inhibitor of HER2, has been proven to target HER2-expressing BCSCs and inhibit the tumor growth of patient-derived xenografts from HER2-negative breast cancer [126].

Given the critical role of TGF- β signaling in EMT and CSC activity, its inhibition has proven to be a promising strategy against drug resistance in chemotherapy [92]. The blocking of TGF-β signaling by a TGF-β type I receptor kinase inhibitor, EW-7197, suppressed paclitaxel-induced EMT and CSC mammosphere formation, reducing the number of lung metastases and increasing survival time in vivo [127]. Moreover, the cross-talk between the TGF-β pathway and Notch signaling in BCSCs has been demonstrated [128,129]. Currently, many different inhibitors of TGF-β pathway are being tested. One of the main is the TGF-β type I RTK inhibitor galunisertib/LY2157299. It has been reported that LY2157299 inhibited CSC expansion induced by paclitaxel alone in TNBC cell lines and in mouse xenografts [92]. Moreover, LY2157299 prevented tumor regrowth after paclitaxel treatment [92]. The evidence of a cardiovascular toxicity of LY2157299 has emerged in some preclinical studies [130,131], but now with the appropriate administration protocols, this issue has been overcome [132]. Some clinical trials are studying the efficacy of LY2157299 but none of these trials is explicitly referring to its anti-CSC activity (Table 2).

Disulfiram, used for chronic alcoholism treatment, is a dithiocarbamate that acts as an inhibitor of ALDH activity [133]. It has been reported that disulfiram inhibited TGF-β-induced EMT and CSC markers in breast cancer [134].

In addition, antibodies targeting clusterin, a stress-activated and apoptosis-associated molecular chaperone also overexpressed in breast cancer [135], have been reported to inhibit TGF-β-induced EMT and to reduce lung metastasis in breast cancer models [136,137]. In particular, a humanized anti-clusterin mAb (AB-16B5) has been tested in patients with advanced solid tumors that showed an inhibition of EMT markers in the tumor biopsies after treatment [91] (Table 2).

#### 3.1.8. Angiogenic Signaling Pathway

Angiogenesis is critically required for cancer development and its inhibition by blocking vascular endothelial growth factor (VEGF) with the monoclonal antibody bevacizumab has been studied in the treatment of different types of cancers [138]. However, these studies reported conflicting opinions on its efficacy. In fact, the antiangiogenic therapy has reported to promote BCSC proliferation driven by hypoxia, limiting the efficacy of antiangiogenic drugs [139]. These findings suggest that antiangiogenic drugs should be combined with CSC-targeted therapies to improve cancer patient outcome. BCSC activity can be induced by hypoxia through a hypoxia-inducible factor 1α (HIF-1α) mediated pathway [140,141]. The blocking of HIF-1α by specific inhibitors, such as ganetespib (a second-generation HSP90 inhibitor), has been reported to be effective in counteracting chemoresistance induced by a paclitaxel or gemcitabine treatment, as demonstrated by in vitro and in vivo studies [140,141]. A phase I clinical trial evaluated the efficacy of a ganetespib treatment in trastuzumab-resistant HER2-positive metastatic breast cancer patients, reporting a good tolerability and slowing down of the progression [96] (Table 2). Another hypoxia-related strategy consists of inhibiting HIF-1α- and HIF-2α-dependent expression of AlkB homolog 5 (ALKBH5) leading to the downregulation of NANOG, a pluripotency key gene in CSCs [142].

### 3.2. New Nano and Biotechnologies Applied to BCSC-Targeting Therapies

Nanotechnology could offer potential solutions for the specific targeting of BCSCs. The aim of nanoparticle technology is to promote the delivery of chemotherapeutic drugs to the tumor site using engineered drug-loaded nanoparticles targeting BCSCs. Currently, an ongoing phase I clinical trial is using lyso-thermosensitive liposomal doxorubicin (LTLD, ThermoDox; Celsion Corporation, NJ, USA) to achieve higher local drug concentrations in metastatic HER2-negative breast cancer patients (NCT03749850) [143]. In addition, nanoparticle albumin-bound (nab)-paclitaxel, in association with atezolizumab, has been proven effective in metastatic TNBC patients (NCT02425891) [144]. Moreover, nanoparticles are commonly used as RNA/DNA carriers in order to prevent degradation and ensure the delivery to the tumor site (see Section 3.2.3 on gene-targeted therapies). Several preclinical studies gave promising results using nanoparticles. In particular, salinomycin-loaded PEGylated polymeric micelles have proven effective in targeting BCSCs in vivo more than salinomycin alone [145]. Other strategies are based on the identification, by specific antibodies or ligands loaded onto nanoparticles, of particular receptors overexpressed on BCSCs [145]. For example, an anti-CD44 antibody conjugated to gold nanorod has been used to target and photo-ablate CD44^+^ subpopulations from three-dimensional MCF-7 mammospheres [146]. Promising results come from the use of salinomycin-loaded nanoparticles coated with hyaluronan (HA), a ligand of CD44. The treatment with these nanoparticles has proven to increase the cellular uptake and specifically target BCSCs [147]. Recently, Han et al. reported the efficacy of HA-conjugated liposomes loaded with gemcitabine in killing BCSCs with a lower systemic toxicity compared with the drug alone in experimental models [148]. Different strategies for HA targeting are under consideration; in particular, the use of small HA oligosaccharides competing with an endogenous HA polymer [149] and the use of antibodies blocking the HA-binding site of CD44 [150] have proven efficacious.

#### 3.2.1. Immunomodulatory Therapy

The genetic engineering of autologous T lymphocytes or dendritic cells (DCs) and cancer vaccines (anti-CSCs or associated-individual proteins) represent promising strategies to elicit a specific antitumor immune response against cancer [151].

The high levels of lymphocytic infiltration is significantly associated with a more-favorable prognosis in patients with early stage TNBC and HER2-positive breast cancer [152]. This infiltration indicates a host antitumor immune response, fundamental for the survival outcome. Recent trials have shown that the targeting of the PD-1 and PD-L1 axis was also clinically effective in metastatic TNBC [152]. In particular, the transduction of T-cell with siRNA against PD-1 ligands or a PD-1/CD28 fusion receptor represents a novel immunotherapy application in breast cancer [153,154].

Moreover, T-cells can be genetically modified to express a novel T cell receptor (TCR) or a chimeric antigen receptor (CAR) that specifically recognize a tumor-associated antigen, inducing the cytolysis of the target cell [155]. However, some limitations are due to the downregulation of HLA class I (antigen presentation) and the required compatibility between patient’s HA haplotype and the TCR [155]. CAR-T cells are engineered T-cells able to target a specific tumor protein expressing chimeric receptors (CARs) that combine both antigen-binding sites and T-cell activating functions by intracellular signaling motifs [155]. This system is independent from HLA and can recognize many targets other than peptides [156].

DCs, involved in the antigen processing and presentation, are currently exploited for their potential antitumor activity. In particular, some studies reported the genetic engineering of tumor cells and their fusion with DCs [157,158]. In addition, DCs can be loaded with tumor antigens or peptides and transfected with tumor-derived RNA or DNA [157,158]. In breast cancer, DCs expressing breast cancer antigens and transfected with siRNA against indoleamine 2,3-dioxygenase have been proven effective in reducing tumor growth and increasing survival in mouse models [159,160].

Therapeutic vaccines are able to stimulate a reactive and competent immune response against tumor antigens. Vaccines can be derived from whole tumor cell lysates, proteins, peptides, DNA, or DCs [151]. Immune cells are able to affect CSCs in vitro and are promising candidates for new strategies in breast cancer immunotherapy [151]. Currently, whole-cell vaccines showed inconsistency regarding the clinical efficacy in cancer patients [161]. Several vaccinations against anti-CSC individual proteins have been investigated in breast cancer (Table 2). In a preclinical study, vaccination against sodium-independent cystine-glutamate antiporter (xCT), the functional subunit of the cysteine/glutamate antiporter system xc-, has been proven effective in the inhibition of mammosphere formation, xenograft growth, and metastasis in EGFR-positive breast cancer cells that overexpressed xCT [162].

#### 3.2.2. Cell-Based Therapy

Different and conflicting data from the literature indicate that MSCs can promote tumor growth and progression through their ability to home in on the tumor microenvironment [163]. It is thanks to this capacity that preclinical studies have suggested MSCs as anti-cancer drug delivery carriers [164,165,166]. In particular, it has been reported that MSCs can uptake and subsequently slowly release paclitaxel through exosomes, inhibiting the proliferation of different cancer cells [164,165,166,167,168,169]. As reported by Scioli et al., adipose-derived stem cells (ASCs) can uptake and release paclitaxel inhibiting CG5 breast cancer survival and proliferation, with no effects on ASC viability and cell cycle [170]. Moreover, it has been demonstrated that gold nanorod embedded hollow periodic mesoporous organosilica nanospheres (GNR@HPMOs) possess high paclitaxel-loading capability, excellent photothermal transfer ability upon near-infrared (NIR) light irradiation, and are well-retained by MSCs after internalization without affecting their viability and tumor-homing capability [171]. Some experiments revealed that GNR@HPMOs-paclitaxel loaded MSCs showed synergistic chemo-photothermal killing effects on breast cancer cells in vitro and in vivo [171]. Genetically engineered ASCs overexpressing TNFα were able to induce apoptosis, via caspase 3/7 activation, in human breast cancer cells and melanoma xenografts [172]. However, there are several limitations, principally regarding safety, that currently prevent the application of MSC-based strategies in clinical trials.

#### 3.2.3. Gene-Targeted Therapies

Currently, therapeutic small interfering RNAs (siRNAs) and microRNAs (miRNAs) represent potential tools for specific gene targeting. Many preclinical studies have been carried out to explore their efficacy in cancer therapy [173]. Commonly, nanoparticles are used as carriers in order to prevent RNA degradation and ensure the delivery to the tumor site [174]. Several siRNA-based therapeutics are in use in cancer patients, instead of miRNA applications, which are still in the preclinical stage. In 2008, the first targeted delivery of siRNA was accomplished in humans. A phase I trial using the siRNA CALAA-01 showed an inhibition of tumor growth by targeting ribonucleotide reductase in patients with advanced solid tumors (Table 2). The nanoparticles, which contained the siRNA, was made of a cyclodextrin-based polymer, a human transferrin protein (TF)-targeting ligand to engage TF receptors on the surface of the cancer cells and a hydrophilic polymer (polyethylene glycol, PEG) used to promote nanoparticle stability in biological fluids. Instrumental analysis confirmed the presence of nanoparticles in the tumor site with low toxicity [109,110].

Atu027 is a siRNA directed against serine/threonine-protein kinase N3 (PKN3), an angiogenic regulator expressed in the vascular endothelium [111]. The siRNA, formulated as liposomal particles (AtuPLEX18), is currently used in an ongoing phase I study in patients with advanced refractory solid tumors, including breast cancer, and has been proven effective as an anti-tumor agent [111].

Only one phase I/II clinical trial has been performed using a liposomal miR-34a mimic, MRX34, which showed antitumor activity in patients with advanced solid tumors, including breast cancer [112]. miR-34 is a tumor suppressor and it has been found to be lost or repressed in cancer patients [175]. It has been reported that miRNAs are involved in the post-transcriptional regulation of breast-cancer-related genes [176]. Antagomirs are small synthetic oligonucleotides involved in the silencing of endogenous miRNAs. The treatment with the anti-miR21 antagomir of MDA-MB-231 cells resulted in the reversion of the EMT and CSC phenotypes [177]. In addition, anti-miR10b antagomirs prevented metastasis formation in a mouse mammary tumor model [178].

Small hairpin RNA lentivirus (shRNA) particles for CD44 knockdown in BCSCs have been proven to induce differentiation into non-CSCs with a lower aggressiveness [179]. It has been reported that autophagy, the lysosomal degradation of cellular components, is involved in the survival and maintenance of BCSCs [180]. The knockdown of autophagy specific genes increased the expression of CD24 and the epithelial-like CD44^+^/CD24^+^ phenotype [180]. However, a study by Kumar et al. reported that the induction of early stage autophagy triggered apoptosis in CD44^+^/CD24^−^ BCSCs and the inhibition of autophagosome formation prevented this phenomenon [181].

The use of shRNA for ganglioside GD3 synthase reduced the CSC population and CSC-associated markers in breast cancer cell lines and completely hindered tumor formation in vivo [182]. OncoGenex Technologies Inc. and Isis Pharmaceuticals Inc. developed OGX-011, a clusterin-inhibiting antisense oligonucleotide, a potential sensitizer of solid tumors that are resistant to conventional cancer therapeutics. A phase II clinical trial of OGX-011 in combination with chemotherapeutic drugs is underway for breast cancer patients [113].

### 3.3. Other Therapeutic Approaches

Salinomycin, an ionophore antibiotic, has been proven effective in eliminating BCSCs in different breast cancer histotypes, likely by autophagy [183], increasing metastasis-free survival and overall survival, as well as inhibiting mammosphere formation and EMT in vitro [147,184]. Combined treatments with salinomycin, conventional drugs (i.e., doxorubicin or paclitaxel), anti-HER2 targeted therapies (monoclonal antibody trastuzumab and lapatinib), and histone deacetylase inhibitors synergistically counteracted tumor growth [185,186]. In particular, the histone deacetylase inhibitor abexinostat has been proven to promote CSC differentiation in breast cancer cell lines with low X-inactive specific transcript expression [187].

Other strategies involve the ALDH activity in combination with conventional therapies to improve breast cancer patients’ outcomes. Croker et al. reported that the inhibition of ALDH activity, by all-trans retinoic acid (ATRA) or diethylaminobenzaldehyde (the specific ALDH inhibitor), counteracted the resistance to chemotherapy (doxorubicin/paclitaxel) and radiotherapy in TNBC cells [188]. In particular, the treatment with ATRA, an inducer of cell differentiation, has been proven to be effective in inhibiting BCSCs [189], but currently, its clinical application (in combination with paclitaxel) has not been successful [190] as an inhibitor. The inhibition of the DNA repair enzyme poly adenosine diphosphate ADP ribose polymerase by Olaparib has proven effective in counteracting CSC activity in breast cancer cells by ERK signaling [191]. It has been reported that the combination with the common chemotherapeutic drug irinotecan induced a decrease in the number of CSCs [191]. Consequently, Olaparib has been proposed as a candidate for the treatment of non-BRCA-related breast cancer [191]. The expression of ATP-binding cassette (ABC) transporters is higher in stem cells compared with normal cells, suggesting a potential role in drug resistance [192,193]. The combined use of dofequidar, an ABC transporter inhibitor, with other chemotherapeutic agents, such as cyclophosphamide, doxorubicin and fluorouracil, demonstrated promising results in patients with advanced or recurrent breast cancer [194]. Dofequidar increased the sensitivity of CSC-like side population cells from different cancer cell lines to anticancer drugs [195]. As described above, conventional chemotherapeutic drugs are not able to target CSCs because of their quiescent state. Therefore, a possible therapeutic strategy is to force CSCs to re-enter the cell cycle, as reported by Gasca et al. [196]. In particular, fbxw7 (F-box protein), a subunit of the stem cell factor SCF-type ubiquitin ligase complex, seems to maintain cell quiescence by reducing the expression of the c-Myc transcriptional factor responsible for the control of the cell cycle and proliferation [197]. Gasc et al. silenced fbxw7 in paclitaxel-resistant TNBC (MDA-MB-468), resensitizing cells to the chemotherapeutic drug item [196]. Moreover, radioresistant BCSCs expressed high levels of ataxia telangiectasia mutated (ATM) (DNA damage surveillance/repair system), and the treatment with an ATM inhibitor has been proven efficacious in re-sensitizing BCSCs to radiation [198].

Finally, different dietary polyphenols seem to affect CSC self-renewal and survival pathways. Among them, sulforaphane from cruciferous vegetables [199,200], epigallocatechin-3-gallate in green tea [201,202], resveratrol [203,204], curcumin [205], and piperine [205] have been reported effective in counteracting BCSC functions.

## 4. Conclusions

Important evidence supports the pivotal role of tumor-initiating or cancer stem cells in anticancer drug resistance and recurrence. Many efforts have been made in the isolation and characterization of breast cancer stem cells (BCSCs), as well as in the identification of possible markers to specifically target this cell population. Several targets have been proposed for the development of BCSC-directed therapies; however, a combination approach directed toward multiple and different pharmacological targets is the most promising. Unfortunately, the outcome of the applied therapeutic methodologies is inconsistent because of the difficulty, by the current markers, to identify a single small population of cells with a high plasticity. The heterogeneity of the BCSC population sustains the therapeutic strategy based on the combination of multiple targets. In addition, the research of new circulating markers for monitoring the effect of anti-BCSC agents is constantly at work. Nano- and biotechnologies associated with gene-targeted strategies represent a promising approach in the development of efficacious drugs targeting CSCs and are able to improve breast cancer therapies.

## Figures and Tables

**Table 1:** CTC-targeting strategies for breast cancer prognosis.

**Epithelial(E)-CTC measurement through Ep-CAM-based systems**									
**Study Design**	**Study Population**	**Patients**	**Patients Positive for CTCs (%)**	**CTC Cut-Off**	**Overall Survival**	**Progression-Free Survival**	**Disease-Free Survival**	**Notes**	**Ref.**
Prospective multicentric study	Metastatic breast cancer	177	87 (49%)	≥5 CTCs/ 7.5 mL of PB	>18 months CTC-negative group vs. 10.1 months CTC positive group *p* < 0.001	7.0 months CTC negative vs. 2.7 months CTC positive, *p* < 0.001	N.R.	First validation study which established the positive-threshold value for the CTC count	[30]
Retrospective multicentric study	Metastatic breast cancer	1944 (911 positive for CTCs)	911 (46.9%)	≥5 CTCs/ 7.5 mL of PB	HR 2.78 for CTC-positive group (95% CI 2.42–3.19, *p* < 0.001)	HR 1.92 for CTC-positive group (95% CI 1.73–2.14, *p* < 0.0001)	N.R.	A positive CTC-count had a significant prognostic value also at 3-5 weeks after the baseline count and at 6-8 week after the first treatment dose. CTC count improved the predictive value of the full clinicopathological prognostic model	[31]
Retrospective multicentric study	Non-metastatic breast cancer (Stage I to III)	3173	640 (20.2%)	≥1 CTC/ 7.5 mL of PB	HR 1.97 for CTC-positive group (95% CI, 1.51 to 2.59 *p* < 0.001)	N.R.	HR, 1.82 for CTC-positive group (95% CI), 1.47 to 2.26	In non-metastatic breast cancer patients, CTC count was confirmed as an independent prognostic factor	[32]
Meta-analysis	Stage I to IV breast cancer	550	N.A.	≥1 HER2/neu positive CTC/ 7.5 mL of PB	In patients without metastasis, Her2-positive CTCs associated with HR 2.273 (95% CI: 1.340–3.853, *p* = 0.002)	In patients without metastasis, HER2/neu-positive CTCs associated with HR = 2.870 (95% CI: 1.298–6.343, *p* = 0.009)	N.R.	HER2/neu-positive CTCs were associated with worse OS and PFS in non-metastatic patients only (non-significant in metastatic patients). This was independent from the HER2/neu status of the primitive tumor	[42]
**Non-Ep-CAM-based systems (measuring both epithelial (E)-CTCs, biphenotypic epithelial/mesenchymal (EM)-CTCs, and mesenchymal (M)-CTCs**									
**Study Design**	**Study Population**	**Patients**	**Patients Positive for CTCs (%)**	**CTC Cut-Off**	**Overall Survival**	**Progression-Free Survival**	**Disease-Free Survival**	**Notes**	**Ref.**
Prospective observational study	Metastatic breast cancer	56	47 (83%)	N.A.	HR 1.035 for EM-CTC positive patients (95% CI, 1.013 to 1.057 *p* = 0.0016) HR 1.019 for E-CTC positive patients (95% CI, 1.004 to 1.034 *p* = 0.0013)	HR 1.021 for EM-CTC positive patients (95% CI 1.004–1.039 *p* = 0.016)	N.R.	Different sub-populations of CTCs were evaluated. Expression of both epithelial and mesenchymal markers was associated to a reduced OS and PFS. CTCs negative for both epithelial and mesenchymal markers were associated with CNS metastases	[53]
Prospective, randomized, open-labeled phase III study	HER2-negative metastatic breast cancer	108	90 (83.3%)	CTCs ≥ 10/ 5 mL PB with a proportion of M-CTCs > 10.7%	N.R.	6.2 months for patients with ≥ 10 CTCs and with a proportion of M-CTCs > 10.7% vs. 9.9 months for the other groups (*p* = 0.010)	Non-significant	Validation study for the CanPatrol CTC enrichment technique. All the three sub-populations of CTCs were evaluated. The follow-up was of 12 months	[54]

Abbreviations: Ref.—References; CTC(s)—circulating tumor cell(s); PB—peripheral blood; HR—hazard ratio; N.R.—Not Reported; N.A.—Not Applicable.

**Table 2:** List of BCSC-targeted strategies used in clinical trials.

Strategy	Target	Drug	Phase	Status	Stage/Type	Identifier	Reference
**Notch signaling**	γ-Secretase	MK-0752	Pilot-study	Unknown	Early stage BC	NCT00756717	[82]
			I/II	Completed	Advanced or metastatic	NCT00645333	[24]
			I	Completed	Metastatic or advanced	NCT00106145	[73]
		PF-03084014 (Nirogacestat)	I	Completed	Advanced	NCT01876251	[83]
			II	Completed	Advanced	NCT02299635	[84]
		LY3039478 (Crenigacestat)	I	Recruiting	Advanced or metastatic	NCT02784795	[85]
		RO4929097 (RG-4733)	I	Completed	Advanced	NCT01208441	[82]
			I	Completed	Advanced	NCT01238133	[86]
			I	Completed	Metastatic	NCT01071564	[76]
			I	Completed	Advanced or metastatic	NCT01149356	[86]
			II	Completed	Advanced, metastatic or recurrent	NCT01151449	[82]
			I	Completed	Refractory	NCT01158274	[82]
			I	Completed	Advanced	NCT01131234	[87]
	Protein-protein interaction	CB-103	I/II	Recruiting	Advanced or metastatic	NCT03422679	[88]
**Hedgehog signaling**	Hedgehog/PTCH1	GDC-0449 (vismodegib)	II	Recruiting	TNBC	NCT02694224	[88]
		LDE225 (sonidegib)	I	Unknown	Advanced	NCT02027376	[88]
			I	Completed	Metastatic	NCT01576666	[89]
**HDAC signaling**	HDAC	Vorinostat	I/II	Terminated	Advanced	NCT01118975	[90]
**HER2 signaling**	HER2	Lapatinib Ditosylate	II	Recruiting	Advanced or metastatic	NCT01868503	-
		Lapatinib	II	Ongoing, not recruiting	Advanced or metastatic	NCT00524303	[25]
**TGF-βIR signaling**	Clusterin	Anti-clusterin mAb AB-16B5	I	Completed	Advanced	NCT02412462	[91]
	TGF-βIR	Galunisertib/LY2157299	II	Completed	Metastatic	NCT02538471	[92]
**PI3K/Akt signaling**	mTOR	Everolimus (RAD001)	III	Completed	Advanced	NCT00863655	[93]
	Akt	MK2206	I	Ongoing, not recruiting	Advanced	NCT01281163	[94]
**Ephrin signaling**	EFNA4	PF-06647263	I	Completed	Advanced	NCT02078752	[95]
**VEGF signaling**	HSP90	Ganetespib	I	Completed	Metastatic HER2^+^	NCT02060253	[96]
**CXCR signaling**	CXCR	Reparixin	II	Recruiting	Metastatic	NCT01861054	[97]
	CXCR	Reparixin	I	Ongoing, not recruiting	Metastatic	NCT02001974	[73]
**Wnt signaling**	PORCN	LGK-974 (WNT974)	I	Recruiting	TNBC	NCT01351103	[98]
	Wnt-5a mimic	Foxy-5	I	Completed	Metastatic	NCT02020291	[99]
				Recruiting	Metastatic	NCT02655952	[100]
	FZD receptors	OMP-18R5 (ventictumab)	I	Completed	Metastatic	NCT01973309	[101]
	ROR1	UC-961 (Cirmtuzumab)	I	Not recruiting	Metastatic	NCT02776917	-
**Immunomodulation**	CSC	CSC whole	I/II	Completed	Advanced	NCT02063893	[26]
	Mammoglobin-A	Vaccination with a pasmid DNA encoding mammaglobin-A	I	Completed	Metastatic	NCT00807781	[102]
		Vaccination with a plasmid DNA encoding mammaglobin-A	I	Recruiting	Advanced	NCT02204098	[103]
	CYP1B1	Vaccination with a plasmid DNA encoding CYP1B1 encapsulated in biodegradable microparticles	I	Completed	Advanced	NCT00381173	-
	Multiple antigens	Plasmid-based vaccination strategy targeting multiple antigens of cancer stem cells	I	Recruiting	Advanced	NCT02157051	-
	IGFBP2, HER2 and IGF1R	Vaccination with a plasmid encoding IGFBP2, HER2 and IGF1R	I	Recruiting	Advanced	NCT02780401	-
	HER2	Vaccination with a plasmid DNA encoding HER2	I	Ongoing	Advanced	NCT00436254	-
	Polypitopes DNA	Personalized polyepitope DNA vaccine	I	Recruiting	Advanced	NCT02348320	-
	RNA vaccines	Immunogenic RNA vaccines	I	Recruiting	Advanced	NCT02316457	-
	HER peptide (NeuVax)	Vaccination with a HER2-targeted peptide (NeuVax)	III	Ongoing	Advanced	NCT01479244	
	Peptide (GP2 and AE37)	Vaccination with a HER2-targeted peptide (GP2 and AE37)	II	Ongoing	Advanced	NCT00524277	[104]
	Synthetic long peptide	Vaccination with personalized synthetic long peptide vaccine	I	Recruiting	Advanced	NCT02427581	-
	Alpha peptide	Vaccination with folate receptor alpha peptide vaccine	I	Recruiting	Advanced	NCT02593227	-
	Peptides tumor-associated	Vaccination with four tumor-associated peptides	I	Recruiting	Rdvanced	NCT02826434	-
	GM-CSF	Vaccination with autologous or allogeneic breast cancer cells engineered to secrete GM-CSF	I	Ongoing	Metastatic	NCT00399529 NCT00317603	-
	TP53	Anti-TP53 TCR-gene engineered lymphocytes and autologous dendritic cell-adenovirus TP53 vaccine	I	Completed	Metastatic	NCT00704938	-
		Recombinant fusion protein of IL-2 linked to a single-chain TCR domain targeting TP53	I	Completed	Metastatic	NCT00496860	-
	Tumor antigen	Immunotherapy with modified TCR targeting CEA tumor antigen	I	Ongoing, recruiting	Metastatic	NCT01022138 NCT02349724	[105]
	Mesothelin	CAR-T cells targeting mesothelin	I	Recruiting	Advanced	NCT0258074 NCT02792114	-
	CD133	CAR-T cells targeting CD133	I	Recruiting	Advanced	NCT02541370	-
	EpCAM	CAR-T cells targeting EpCAM	I	Recruiting	Recurrent	NCT02915445	-
	ROR1	CAR-T cells targeting ROR1	I	Recruiting	Recurrent	NCT02706392	[106]
	MUC-1	CAR-T cells targeting MUC-1	I/II	Recruiting	Advanced	NCT02587689	-
	HER2	CAR-T cells targeting HER2	I/II	Recruiting	Recurrent	NCT02547961 NCT02713984	-
	MET	CAR-T cells targeting MET proto-oncogene	I/II	Ongoing	Advanced	NCT01837602	[107]
	TP53	Vaccination with adenovirus-TP53 trasduced DCs	I/II	Ongoing	Recurrent and advanced	NCT01042535	[108]
	HER2	Vaccination with adenovirus-HER2- trasduced DCs	I	Completed	Advanced	NCT00197522 NCT01730118	-
	Cyclin B1/WT-1/CEF	Vaccination cyclin B1/WT-1/CEF pool-loaded DCs	I	Recruiting	Advanced	NCT02018458	-
	Onco-peptides	Vaccination with autologous DCs pulsed with onco-peptides	I	Completed	Metastatic	NCT00197925	-
	Tumor blood vessel antigen -derived peptides	Vaccination with DCs incorporating tumor blood vessel antigen-derived peptides	I	Recruiting	Metastatic	NCT02479230	-
	Frizzled (Fzd) receptor	Vantictumab/OMP-18R5 Anti-Frizzled receptors mAb	Ib	Completed	Metastatic	NCT01973309	-
**Gene-targeting**	RRM2	CALAA-01 (transferin-targeted cyclodextrin-containing polymer carrying siRNA against RRM2	I	Completed	Advanced	NCT00689065	[109,110]
	Protein kinase N3 gene	Lipoplexed Atu-027 (AtuPLEX)	I	Recruiting	Advanced	NCT00938574	[111]
	MiR-34a	MRX34 liposomal miR-34a mimic	I	Completed	Advanced	NCT01829971	[112]
	Clusterin	OGX01 antisense oligonucleotide	II	Completed	Metastatic	NCT01578655	[113]

## References

[1] WHO (2018). Breast Cancer. https://www.who.int/cancer/prevention/diagnosis-screening/breast-cancer/en/.

[2] Vargo-Gogola T., Rosen J.M. (2007). Modelling breast cancer: One size does not fit all. Nat. Rev. Cancer.

[3] Koren S., Bentires-Alj M. (2015). Breast Tumor Heterogeneity: Source of Fitness, Hurdle for Therapy. Mol. Cell.

[4] Al-Hajj M., Wicha M.S., Benito-Hernandez A., Morrison S.J., Clarke M.F. (2003). Prospective identification of tumorigenic breast cancer cells. Proc. Natl. Acad. Sci. USA.

[5] Lawson J.C., Blatch G.L., Edkins A.L. (2009). Cancer stem cells in breast cancer and metastasis. Breast Cancer Res. Treat..

[6] Baccelli I., Schneeweiss A., Riethdorf S., Stenzinger A., Schillert A., Vogel V., Klein C., Saini M., Bauerle T., Wallwiener M. (2013). Identification of a population of blood circulating tumor cells from breast cancer patients that initiates metastasis in a xenograft assay. Nat. Biotechnol..

[7] Creighton C.J., Li X., Landis M., Dixon J.M., Neumeister V.M., Sjolund A., Rimm D.L., Wong H., Rodriguez A., Herschkowitz J.I. (2009). Residual breast cancers after conventional therapy display mesenchymal as well as tumor-initiating features. Proc. Natl. Acad. Sci. USA.

[8] Ginestier C., Hur M.H., Charafe-Jauffret E., Monville F., Dutcher J., Brown M., Jacquemier J., Viens P., Kleer C.G., Liu S. (2007). ALDH1 is a marker of normal and malignant human mammary stem cells and a predictor of poor clinical outcome. Cell Stem Cell.

[9] Takahashi-Yanaga F., Kahn M. (2010). Targeting Wnt signaling: Can we safely eradicate cancer stem cells?. Clin. Cancer Res..

[10] Smit L., Berns K., Spence K., Ryder W.D., Zeps N., Madiredjo M., Beijersbergen R., Bernards R., Clarke R.B. (2016). An integrated genomic approach identifies that the PI3K/AKT/FOXO pathway is involved in breast cancer tumor initiation. Oncotarget.

[11] Woosley A.N., Dalton A.C., Hussey G.S., Howley B.V., Mohanty B.K., Grelet S., Dincman T., Bloos S., Olsen S.K., Howe P.H. (2019). TGFbeta promotes breast cancer stem cell self-renewal through an ILEI/LIFR signaling axis. Oncogene.

[12] Zhang L., Ridgway L.D., Wetzel M.D., Ngo J., Yin W., Kumar D., Goodman J.C., Groves M.D., Marchetti D. (2013). The identification and characterization of breast cancer CTCs competent for brain metastasis. Sci. Transl. Med..

[13] Liu H., Patel M.R., Prescher J.A., Patsialou A., Qian D., Lin J., Wen S., Chang Y.F., Bachmann M.H., Shimono Y. (2010). Cancer stem cells from human breast tumors are involved in spontaneous metastases in orthotopic mouse models. Proc. Natl. Acad. Sci. USA.

[14] Charafe-Jauffret E., Ginestier C., Iovino F., Wicinski J., Cervera N., Finetti P., Hur M.H., Diebel M.E., Monville F., Dutcher J. (2009). Breast cancer cell lines contain functional cancer stem cells with metastatic capacity and a distinct molecular signature. Cancer Res..

[15] Ksiazkiewicz M., Markiewicz A., Zaczek A.J. (2012). Epithelial-mesenchymal transition: A hallmark in metastasis formation linking circulating tumor cells and cancer stem cells. Pathobiology.

[16] Lamouille S., Xu J., Derynck R. (2014). Molecular mechanisms of epithelial-mesenchymal transition. Nat. Rev. Mol. Cell Boil..

[17] Mani S.A., Guo W., Liao M.J., Eaton E.N., Ayyanan A., Zhou A.Y., Brooks M., Reinhard F., Zhang C.C., Shipitsin M. (2008). The epithelial-mesenchymal transition generates cells with properties of stem cells. Cell.

[18] Del Pozo Martin Y., Park D., Ramachandran A., Ombrato L., Calvo F., Chakravarty P., Spencer-Dene B., Derzsi S., Hill C.S., Sahai E. (2015). Mesenchymal Cancer Cell-Stroma Crosstalk Promotes Niche Activation, Epithelial Reversion, and Metastatic Colonization. Cell Rep..

[19] Yu M., Bardia A., Wittner B.S., Stott S.L., Smas M.E., Ting D.T., Isakoff S.J., Ciciliano J.C., Wells M.N., Shah A.M. (2013). Circulating breast tumor cells exhibit dynamic changes in epithelial and mesenchymal composition. Science.

[20] Liu S., Cong Y., Wang D., Sun Y., Deng L., Liu Y., Martin-Trevino R., Shang L., McDermott S.P., Landis M.D. (2014). Breast cancer stem cells transition between epithelial and mesenchymal states reflective of their normal counterparts. Stem Cell Rep..

[21] Yang F., Xu J., Tang L., Guan X. (2017). Breast cancer stem cell: The roles and therapeutic implications. Cell. Mol. Life Sci. CMLS.

[22] Luo Y.T., Cheng J., Feng X., He S.J., Wang Y.W., Huang Q. (2018). The viable circulating tumor cells with cancer stem cells feature, where is the way out?. J. Exp. Clin. Cancer Res. CR.

[23] Hernandez-Vargas H., von Kobbe C., Sanchez-Estevez C., Julian-Tendero M., Palacios J., Moreno-Bueno G. (2007). Inhibition of paclitaxel-induced proteasome activation influences paclitaxel cytotoxicity in breast cancer cells in a sequence-dependent manner. Cell Cycle.

[24] Aktas B., Tewes M., Fehm T., Hauch S., Kimmig R., Kasimir-Bauer S. (2009). Stem cell and epithelial-mesenchymal transition markers are frequently overexpressed in circulating tumor cells of metastatic breast cancer patients. Breast Cancer Res. BCR.

[25] Holmes F.A., Espina V., Liotta L.A., Nagarwala Y.M., Danso M., McIntyre K.J., Osborne C.R., Anderson T., Krekow L., Blum J.L. (2013). Pathologic complete response after preoperative anti-HER2 therapy correlates with alterations in PTEN, FOXO, phosphorylated Stat5, and autophagy protein signaling. BMC Res. Notes.

[26] Ning N., Pan Q., Zheng F., Teitz-Tennenbaum S., Egenti M., Yet J., Li M., Ginestier C., Wicha M.S., Moyer J.S. (2012). Cancer stem cell vaccination confers significant antitumor immunity. Cancer Res..

[27] Chiotaki R., Polioudaki H., Theodoropoulos P.A. (2015). Cancer stem cells in solid and liquid tissues of breast cancer patients: Characterization and therapeutic perspectives. Curr. Cancer Drug Targets.

[28] Kalluri R. (2009). EMT: When epithelial cells decide to become mesenchymal-like cells. J. Clin. Investig..

[29] Allard W.J., Matera J., Miller M.C., Repollet M., Connelly M.C., Rao C., Tibbe A.G., Uhr J.W., Terstappen L.W. (2004). Tumor cells circulate in the peripheral blood of all major carcinomas but not in healthy subjects or patients with nonmalignant diseases. Clin. Cancer Res..

[30] Cristofanilli M., Budd G.T., Ellis M.J., Stopeck A., Matera J., Miller M.C., Reuben J.M., Doyle G.V., Allard W.J., Terstappen L.W. (2004). Circulating tumor cells, disease progression, and survival in metastatic breast cancer. N. Engl. J. Med..

[31] Bidard F.C., Peeters D.J., Fehm T., Nole F., Gisbert-Criado R., Mavroudis D., Grisanti S., Generali D., Garcia-Saenz J.A., Stebbing J. (2014). Clinical validity of circulating tumour cells in patients with metastatic breast cancer: A pooled analysis of individual patient data. Lancet Oncol..

[32] Janni W.J., Rack B., Terstappen L.W., Pierga J.Y., Taran F.A., Fehm T., Hall C., de Groot M.R., Bidard F.C., Friedl T.W. (2016). Pooled Analysis of the Prognostic Relevance of Circulating Tumor Cells in Primary Breast Cancer. Clin. Cancer Res..

[33] Bidard F.-C., Jacot W., Dureau S., Brain E., Bachelot T., Bourgeois H., Goncalves A., Ladoire S., Naman H., Dalenc F. (2019). Abstract GS3-07: Clinical utility of circulating tumor cell count as a tool to chose between first line hormone therapy and chemotherapy for ER + HER2-metastatic breast cancer: Results of the phase III STIC CTC trial. Cancer Res..

[34] Ignatiadis M., Rack B., Rothe F., Riethdorf S., Decraene C., Bonnefoi H., Dittrich C., Messina C., Beauvois M., Trapp E. (2016). Liquid biopsy-based clinical research in early breast cancer: The EORTC 90091-10093 Treat CTC trial. Eur. J. Cancer.

[35] Bidard F.-C., Dubot C., Venat-Bouvet L., Lortholary A., Bourgeois H., Bollet M., Servent Hanon V., Luporsi-Gely E., Espie M., Guiu S. (2017). 117P-Anti-HER2 therapy efficacy in HER2-negative metastatic breast cancer with HER2-amplified circulating tumor cells: Results of the CirCe T-DM1 trial. Ann. Oncol..

[36] Hayes D.F., Cristofanilli M., Budd G.T., Ellis M.J., Stopeck A., Miller M.C., Matera J., Allard W.J., Doyle G.V., Terstappen L.W. (2006). Circulating tumor cells at each follow-up time point during therapy of metastatic breast cancer patients predict progression-free and overall survival. Clin. Cancer Res..

[37] Boral D., Vishnoi M., Liu H.N., Yin W., Sprouse M.L., Scamardo A., Hong D.S., Tan T.Z., Thiery J.P., Chang J.C. (2017). Molecular characterization of breast cancer CTCs associated with brain metastasis. Nat. Commun..

[38] Kalinsky K., Mayer J.A., Xu X., Pham T., Wong K.L., Villarin E., Pircher T.J., Brown M., Maurer M.A., Bischoff F.Z. (2015). Correlation of hormone receptor status between circulating tumor cells, primary tumor, and metastasis in breast cancer patients. Clin. Transl. Oncol..

[39] Paoletti C., Muniz M.C., Thomas D.G., Griffith K.A., Kidwell K.M., Tokudome N., Brown M.E., Aung K., Miller M.C., Blossom D.L. (2015). Development of circulating tumor cell-endocrine therapy index in patients with hormone receptor-positive breast cancer. Clin. Cancer Res..

[40] Somlo G., Lau S.K., Frankel P., Hsieh H.B., Liu X., Yang L., Krivacic R., Bruce R.H. (2011). Multiple biomarker expression on circulating tumor cells in comparison to tumor tissues from primary and metastatic sites in patients with locally advanced/inflammatory, and stage IV breast cancer, using a novel detection technology. Breast Cancer Res. Treat..

[41] Aktas B., Kasimir-Bauer S., Muller V., Janni W., Fehm T., Wallwiener D., Pantel K., Tewes M. (2016). Comparison of the HER2, estrogen and progesterone receptor expression profile of primary tumor, metastases and circulating tumor cells in metastatic breast cancer patients. BMC Cancer.

[42] Wang C.H., Chang C.J., Yeh K.Y., Chang P.H., Huang J.S. (2017). The Prognostic Value of HER2-Positive Circulating Tumor Cells in Breast Cancer Patients: A Systematic Review and Meta-Analysis. Clin. Breast Cancer.

[43] Oesterreich S., Davidson N.E. (2013). The search for ESR1 mutations in breast cancer. Nat. Genet..

[44] Neves R.P., Raba K., Schmidt O., Honisch E., Meier-Stiegen F., Behrens B., Mohlendick B., Fehm T., Neubauer H., Klein C.A. (2014). Genomic high-resolution profiling of single CKpos/CD45neg flow-sorting purified circulating tumor cells from patients with metastatic breast cancer. Clin. Chem..

[45] Gradilone A., Naso G., Raimondi C., Cortesi E., Gandini O., Vincenzi B., Saltarelli R., Chiapparino E., Spremberg F., Cristofanilli M. (2011). Circulating tumor cells (CTCs) in metastatic breast cancer (MBC): Prognosis, drug resistance and phenotypic characterization. Ann. Oncol..

[46] Wang F., Li Y.C., Liu L.P., Zhang H.M., Tong S. (2016). Circulating Tumor Cells and Tumor Stem Cells Detection in the Peripheral Blood Mononuclear Cells of Breast Cancer. J. Clin. Lab. Anal..

[47] Yan L., Xu F., Dai C.L. (2018). Relationship between epithelial-to-mesenchymal transition and the inflammatory microenvironment of hepatocellular carcinoma. J. Exp. Clin. Cancer Res. CR.

[48] Reuben J.M., Lee B.N., Gao H., Cohen E.N., Mego M., Giordano A., Wang X., Lodhi A., Krishnamurthy S., Hortobagyi G.N. (2011). Primary breast cancer patients with high risk clinicopathologic features have high percentages of bone marrow epithelial cells with ALDH activity and CD44^+^CD24lo cancer stem cell phenotype. Eur. J. Cancer.

[49] Barriere G., Riouallon A., Renaudie J., Tartary M., Rigaud M. (2012). Mesenchymal and stemness circulating tumor cells in early breast cancer diagnosis. BMC Cancer.

[50] Kasimir-Bauer S., Hoffmann O., Wallwiener D., Kimmig R., Fehm T. (2012). Expression of stem cell and epithelial-mesenchymal transition markers in primary breast cancer patients with circulating tumor cells. Breast Cancer Res. BCR.

[51] Markiewicz A., Ksiazkiewicz M., Welnicka-Jaskiewicz M., Seroczynska B., Skokowski J., Szade J., Zaczek A.J. (2014). Mesenchymal phenotype of CTC-enriched blood fraction and lymph node metastasis formation potential. PLoS ONE.

[52] Papadaki M.A., Kallergi G., Zafeiriou Z., Manouras L., Theodoropoulos P.A., Mavroudis D., Georgoulias V., Agelaki S. (2014). Co-expression of putative stemness and epithelial-to-mesenchymal transition markers on single circulating tumour cells from patients with early and metastatic breast cancer. BMC Cancer.

[53] Bulfoni M., Gerratana L., Del Ben F., Marzinotto S., Sorrentino M., Turetta M., Scoles G., Toffoletto B., Isola M., Beltrami C.A. (2016). In patients with metastatic breast cancer the identification of circulating tumor cells in epithelial-to-mesenchymal transition is associated with a poor prognosis. Breast Cancer Res. BCR.

[54] Guan X., Ma F., Li C., Wu S., Hu S., Huang J., Sun X., Wang J., Luo Y., Cai R. (2019). The prognostic and therapeutic implications of circulating tumor cell phenotype detection based on epithelial-mesenchymal transition markers in the first-line chemotherapy of HER2-negative metastatic breast cancer. Cancer Commun..

[55] Marotta L.L., Almendro V., Marusyk A., Shipitsin M., Schemme J., Walker S.R., Bloushtain-Qimron N., Kim J.J., Choudhury S.A., Maruyama R. (2011). The JAK2/STAT3 signaling pathway is required for growth of CD44^+^CD24^−^ stem cell-like breast cancer cells in human tumors. J. Clin. Investig..

[56] Charafe-Jauffret E., Ginestier C., Bertucci F., Cabaud O., Wicinski J., Finetti P., Josselin E., Adelaide J., Nguyen T.T., Monville F. (2013). ALDH1-positive cancer stem cells predict engraftment of primary breast tumors and are governed by a common stem cell program. Cancer Res..

[57] Wang J., Chang S., Li G., Sun Y. (2017). Application of liquid biopsy in precision medicine: Opportunities and challenges. Front. Med..

[58] Cheng C.W., Yu J.C., Hsieh Y.H., Liao W.L., Shieh J.C., Yao C.C., Lee H.J., Chen P.M., Wu P.E., Shen C.Y. (2018). Increased Cellular Levels of MicroRNA-9 and MicroRNA-221 Correlate with Cancer Stemness and Predict Poor Outcome in Human Breast Cancer. Cell. Physiol. Biochem..

[59] Braun M., Markiewicz A., Kordek R., Sadej R., Romanska H. (2019). Profiling of Invasive Breast Carcinoma Circulating Tumour Cells—Are We Ready for the ‘Liquid’ Revolution?. Cancers.

[60] Neumeister V., Agarwal S., Bordeaux J., Camp R.L., Rimm D.L. (2010). In situ identification of putative cancer stem cells by multiplexing ALDH1, CD44, and cytokeratin identifies breast cancer patients with poor prognosis. Am. J. Pathol..

[61] Rabinovich I., Sebastiao A.P.M., Lima R.S., Urban C.A., Junior E.S., Anselmi K.F., Elifio-Esposito S., De Noronha L., Moreno-Amaral A.N. (2018). Cancer stem cell markers ALDH1 and CD44+/CD24− phenotype and their prognosis impact in invasive ductal carcinoma. Eur. J. Histochem. EJH.

[62] Zhong Y., Shen S., Zhou Y., Mao F., Guan J., Lin Y., Xu Y., Sun Q. (2014). ALDH1 is a better clinical indicator for relapse of invasive ductal breast cancer than the CD44+/CD24− phenotype. Med. Oncol..

[63] Li W., Ma H., Zhang J., Zhu L., Wang C., Yang Y. (2017). Unraveling the roles of CD44/CD24 and ALDH1 as cancer stem cell markers in tumorigenesis and metastasis. Sci. Rep..

[64] Gwak J.M., Kim M., Kim H.J., Jang M.H., Park S.Y. (2017). Expression of embryonal stem cell transcription factors in breast cancer: Oct4 as an indicator for poor clinical outcome and tamoxifen resistance. Oncotarget.

[65] Bhatt S., Stender J.D., Joshi S., Wu G., Katzenellenbogen B.S. (2016). OCT-4: A novel estrogen receptor-alpha collaborator that promotes tamoxifen resistance in breast cancer cells. Oncogene.

[66] Liu J.C., Zacksenhouse M., Eisen A., Nofech-Mozes S., Zacksenhaus E. (2017). Identification of cell proliferation, immune response and cell migration as critical pathways in a prognostic signature for HER2+: ERalpha-breast cancer. PLoS ONE.

[67] Pece S., Disalvatore D., Tosoni D., Vecchi M., Confalonieri S., Bertalot G., Viale G., Colleoni M., Veronesi P., Galimberti V. (2019). Identification and clinical validation of a multigene assay that interrogates the biology of cancer stem cells and predicts metastasis in breast cancer: A retrospective consecutive study. EBioMedicine.

[68] Li X., Lewis M.T., Huang J., Gutierrez C., Osborne C.K., Wu M.F., Hilsenbeck S.G., Pavlick A., Zhang X., Chamness G.C. (2008). Intrinsic resistance of tumorigenic breast cancer cells to chemotherapy. J. Natl. Cancer Inst..

[69] Balzer E.M., Whipple R.A., Cho E.H., Matrone M.A., Martin S.S. (2010). Antimitotic chemotherapeutics promote adhesive responses in detached and circulating tumor cells. Breast Cancer Res. Treat..

[70] Rasul S., Balasubramanian R., Filipovic A., Slade M.J., Yague E., Coombes R.C. (2009). Inhibition of gamma-secretase induces G2/M arrest and triggers apoptosis in breast cancer cells. Br. J. Cancer.

[71] Kondratyev M., Kreso A., Hallett R.M., Girgis-Gabardo A., Barcelon M.E., Ilieva D., Ware C., Majumder P.K., Hassell J.A. (2012). Gamma-secretase inhibitors target tumor-initiating cells in a mouse model of ERBB2 breast cancer. Oncogene.

[72] Farnie G., Clarke R.B. (2007). Mammary stem cells and breast cancer—Role of Notch signalling. Stem Cell Rev..

[73] Schott A.F., Landis M.D., Dontu G., Griffith K.A., Layman R.M., Krop I., Paskett L.A., Wong H., Dobrolecki L.E., Lewis M.T. (2013). Preclinical and clinical studies of gamma secretase inhibitors with docetaxel on human breast tumors. Clin. Cancer Res..

[74] Cochrane C.R., Szczepny A., Watkins D.N., Cain J.E. (2015). Hedgehog Signaling in the Maintenance of Cancer Stem Cells. Cancers.

[75] Sims-Mourtada J., Opdenaker L.M., Davis J., Arnold K.M., Flynn D. (2015). Taxane-induced hedgehog signaling is linked to expansion of breast cancer stem-like populations after chemotherapy. Mol. Carcinog..

[76] Ramaswamy B., Lu Y., Teng K.Y., Nuovo G., Li X., Shapiro C.L., Majumder S. (2012). Hedgehog signaling is a novel therapeutic target in tamoxifen-resistant breast cancer aberrantly activated by PI3K/AKT pathway. Cancer Res..

[77] Jang G.B., Hong I.S., Kim R.J., Lee S.Y., Park S.J., Lee E.S., Park J.H., Yun C.H., Chung J.U., Lee K.J. (2015). Wnt/beta-Catenin Small-Molecule Inhibitor CWP232228 Preferentially Inhibits the Growth of Breast Cancer Stem-like Cells. Cancer Res..

[78] Katoh M., Katoh M. (2017). Molecular genetics and targeted therapy of WNT-related human diseases (Review). Int. J. Mol. Med..

[79] Liu J., Pan S., Hsieh M.H., Ng N., Sun F., Wang T., Kasibhatla S., Schuller A.G., Li A.G., Cheng D. (2013). Targeting Wnt-driven cancer through the inhibition of Porcupine by LGK974. Proc. Natl. Acad. Sci. USA.

[80] Hallett R.M., Kondratyev M.K., Giacomelli A.O., Nixon A.M., Girgis-Gabardo A., Ilieva D., Hassell J.A. (2012). Small molecule antagonists of the Wnt/beta-catenin signaling pathway target breast tumor-initiating cells in a Her2/Neu mouse model of breast cancer. PLoS ONE.

[81] Cui B., Zhang S., Chen L., Yu J., Widhopf G.F., Fecteau J.F., Rassenti L.Z., Kipps T.J. (2013). Targeting ROR1 inhibits epithelial-mesenchymal transition and metastasis. Cancer Res..

[82] Venkatesh V., Nataraj R., Thangaraj G.S., Karthikeyan M., Gnanasekaran A., Kaginelli S.B., Kuppanna G., Kallappa C.G., Basalingappa K.M. (2018). Targeting Notch signalling pathway of cancer stem cells. Stem Cell Investig..

[83] Locatelli M.A., Aftimos P., Dees E.C., LoRusso P.M., Pegram M.D., Awada A., Huang B., Cesari R., Jiang Y., Shaik M.N. (2017). Phase I study of the gamma secretase inhibitor PF-03084014 in combination with docetaxel in patients with advanced triple-negative breast cancer. Oncotarget.

[84] Ocana A., Gil-Martin M., Martín M., Rojo F., Antolín S., Guerrero Á., Trigo J.M., Muñoz M., Pandiella A., Diego N.G. (2017). A phase I study of the SRC kinase inhibitor dasatinib with trastuzumab and paclitaxel as first line therapy for patients with HER2-overexpressing advanced breast cancer. GEICAM/2010-04 study. Oncotarget.

[85] McCartney A., Moretti E., Sanna G., Pestrin M., Risi E., Malorni L., Biganzoli L., Di Leo A. (2018). The role of abemaciclib in treatment of advanced breast cancer. Ther. Adv. Med. Oncol..

[86] Strosberg J.R., Yeatman T., Weber J., Coppola D., Schell M.J., Han G., Almhanna K., Kim R., Valone T., Jump H. (2012). A phase II study of RO4929097 in metastatic colorectal cancer. Eur. J. Cancer..

[87] Koury J., Zhong L., Hao J. (2017). Targeting Signaling Pathways in Cancer Stem Cells for Cancer Treatment. Stem Cells Int..

[88] Palomeras S., Ruiz-Martínez S., Puig T. (2018). Targeting Breast Cancer Stem Cells to Overcome Treatment Resistance. Molecules.

[89] Criscitiello C., Viale G., Curigliano G., Goldhirsch A. (2018). Profile of buparlisib and its potential in the treatment of breast cancer: Evidence to date. Breast Cancer.

[90] Lustberg M.B., Ramaswamy B. (2011). Epigenetic Therapy in Breast Cancer. Curr. Breast Cancer Rep..

[91] Ferrario C., Laurin J., Van Kempen L., Lambert C., Spatz A., Markova O., Batist G., Langleben A., Filion M., Jolivet J. (2017). Abstract CT098: Phase 1 first-in-human study of anti-clusterin antibody AB-16B5 in patients with advanced solid malignancies. Cancer Res..

[92] Bhola N.E., Balko J.M., Dugger T.C., Kuba M.G., Sanchez V., Sanders M., Stanford J., Cook R.S., Arteaga C.L. (2013). TGF-beta inhibition enhances chemotherapy action against triple-negative breast cancer. J. Clin. Investig..

[93] Baselga J., Campone M., Piccart M., Burris H.A., Rugo H.S., Sahmoud T., Noguchi S., Gnant M., Pritchard K.I., Lebrun F. (2012). Everolimus in postmenopausal hormone-receptor-positive advanced breast cancer. N. Engl. J. Med..

[94] Alferez D.G., Simões B.M., Howell S.J., Clarke R.B. (2018). The Role of Steroid Hormones in Breast and Effects on Cancer Stem Cells. Curr. Stem Cell Rep..

[95] Garrido-Laguna I., Krop I., Burris H.A., Hamilton E., Braiteh F., Weise A.M., Abu-Khalaf M., Werner T.L., Pirie-Shepherd S., Zopf C.J. (2019). First-in-human, phase I study of PF-06647263, an anti-EFNA4 calicheamicin antibody-drug conjugate, in patients with advanced solid tumors. Int. J. Cancer.

[96] Jhaveri K., Wang R., Teplinsky E., Chandarlapaty S., Solit D., Cadoo K., Speyer J., D’Andrea G., Adams S., Patil S. (2017). A phase I trial of ganetespib in combination with paclitaxel and trastuzumab in patients with human epidermal growth factor receptor-2 (HER2)-positive metastatic breast cancer. Breast Cancer Res. BCR.

[97] Goldstein L.S.J., Perez R., Vito C., Reuben J., Landis M. (2013). Abstract OT2-6-03: A single arm, preoperative, pilot study to evaluate the safety and biological effects of orally administered reparixin in early breast cancer patients who are candidates for surgery. Cancer Res..

[98] Leung E.Y., Askarian-Amiri M.E., Sarkar D., Ferraro-Peyret C., Joseph W.R., Finlay G.J., Baguley B.C. (2017). Endocrine Therapy of Estrogen Receptor-Positive Breast Cancer Cells: Early Differential Effects on Stem Cell Markers. Front. Oncol..

[99] Canesin G., Evans-Axelsson S., Hellsten R., Krzyzanowska A., Prasad C.P., Bjartell A., Andersson T. (2017). Treatment with the WNT5A-mimicking peptide Foxy-5 effectively reduces the metastatic spread of WNT5A-low prostate cancer cells in an orthotopic mouse model. PLOS ONE.

[100] Goldsberry W.N., Londoño A., Randall T.D., Norian L.A., Arend R.C. (2019). A Review of the Role of Wnt in Cancer Immunomodulation. Cancers.

[101] Fischer M.M., Cancilla B., Yeung V.P., Cattaruzza F., Chartier C., Murriel C.L., Cain J., Tam R., Cheng C.Y., Evans J.W. (2017). WNT antagonists exhibit unique combinatorial antitumor activity with taxanes by potentiating mitotic cell death. Sci. Adv..

[102] Tiriveedhi V., Tucker N., Herndon J., Li L., Sturmoski M., Ellis M., Ma C., Naughton M., Lockhart A.C., Gao F. (2014). Safety and preliminary evidence of biologic efficacy of a mammaglobin-a DNA vaccine in patients with stable metastatic breast cancer. Clin. Cancer Res..

[103] Kim S.W., Goedegebuure P., Gillanders W.E. (2016). Mammaglobin-A is a target for breast cancer vaccination. OncoImmunology.

[104] Mittendorf E.A., Ardavanis A., Litton J.K., Shumway N.M., Hale D.F., Murray J.L., Perez S.A., Ponniah S., Baxevanis C.N., Papamichail M. (2016). Primary analysis of a prospective, randomized, single-blinded phase II trial evaluating the HER2 peptide GP2 vaccine in breast cancer patients to prevent recurrence. Oncotarget.

[105] Jagtap B.D., Thakur A., Deol A., Al-Kadhimi Z., Simon M.S., Flaherty L.E. (2014). Phase II trial evaluating HER2 targeted activated T cells in advanced HER2 low expressing breast cancer patients. J. Clin. Oncol..

[106] Specht J.M., Lee S., Turtle C., Berger C., Veatch J., Gooley T., Mullane E., Chaney C., Riddell S., Maloney D.G. (2018). Phase I study of immunotherapy for advanced ROR1+ malignancies with autologous ROR1-specific chimeric antigen receptor-modified (CAR)-T cells. J. Clin. Oncol..

[107] Tchou J., Zhao Y., Levine B.L., Zhang P.J., Davis M.M., Melenhorst J.J., Kulikovskaya I., Brennan A.L., Liu X., Lacey S.F. (2017). Safety and Efficacy of Intratumoral Injections of Chimeric Antigen Receptor (CAR) T Cells in Metastatic Breast Cancer. Cancer Immunol. Res..

[108] Soliman H., Khambati F., Han H.S., Ismail-Khan R., Bui M.M., Sullivan D.M., Antonia S. (2018). A phase-1/2 study of adenovirus-p53 transduced dendritic cell vaccine in combination with indoximod in metastatic solid tumors and invasive breast cancer. Oncotarget.

[109] Davis M.E. (2009). The first targeted delivery of siRNA in humans via a self-assembling, cyclodextrin polymer-based nanoparticle: From concept to clinic. Mol. Pharm..

[110] Davis M.E., Zuckerman J.E., Choi C.H., Seligson D., Tolcher A., Alabi C.A., Yen Y., Heidel J.D., Ribas A. (2010). Evidence of RNAi in humans from systemically administered siRNA via targeted nanoparticles. Nature.

[111] Schultheis B., Strumberg D., Santel A., Vank C., Gebhardt F., Keil O., Lange C., Giese K., Kaufmann J., Khan M. (2014). First-in-human phase I study of the liposomal RNA interference therapeutic Atu027 in patients with advanced solid tumors. J. Clin. Oncol..

[112] Beg M.S., Brenner A.J., Sachdev J., Borad M., Kang Y.K., Stoudemire J., Smith S., Bader A.G., Kim S., Hong D.S. (2017). Phase I study of MRX34, a liposomal miR-34a mimic, administered twice weekly in patients with advanced solid tumors. Investig. New Drugs.

[113] Chia S., Dent S., Ellard S., Ellis P.M., Vandenberg T., Gelmon K., Powers J., Walsh W., Seymour L., Eisenhauer E.A. (2009). Phase II trial of OGX-011 in combination with docetaxel in metastatic breast cancer. Clin. Cancer Res..

[114] Zhang X., Zhang S., Liu Y., Liu J., Ma Y., Zhu Y., Zhang J. (2012). Effects of the combination of RAD001 and docetaxel on breast cancer stem cells. Eur. J. Cancer.

[115] Perez White B.E., Getsios S. (2014). Eph receptor and ephrin function in breast, gut, and skin epithelia. Cell Adhes. Migr..

[116] Hachim I.Y., Villatoro M., Canaff L., Hachim M.Y., Boudreault J., Haiub H., Ali S., Lebrun J.J. (2017). Transforming Growth Factor-beta Regulation of Ephrin Type-A Receptor 4 Signaling in Breast Cancer Cellular Migration. Sci. Rep..

[117] Damelin M., Bankovich A., Park A., Aguilar J., Anderson W., Santaguida M., Aujay M., Fong S., Khandke K., Pulito V. (2015). Anti-EFNA4 Calicheamicin Conjugates Effectively Target Triple-Negative Breast and Ovarian Tumor-Initiating Cells to Result in Sustained Tumor Regressions. Clin. Cancer Res..

[118] Zlotnik A., Yoshie O. (2012). The chemokine superfamily revisited. Immunity.

[119] Waugh D.J., Wilson C. (2008). The interleukin-8 pathway in cancer. Clin. Cancer Res..

[120] Singh J.K., Farnie G., Bundred N.J., Simoes B.M., Shergill A., Landberg G., Howell S.J., Clarke R.B. (2013). Targeting CXCR1/2 significantly reduces breast cancer stem cell activity and increases the efficacy of inhibiting HER2 via HER2-dependent and-independent mechanisms. Clin. Cancer Res..

[121] Bertini R., Allegretti M., Bizzarri C., Moriconi A., Locati M., Zampella G., Cervellera M.N., Di Cioccio V., Cesta M.C., Galliera E. (2004). Noncompetitive allosteric inhibitors of the inflammatory chemokine receptors CXCR1 and CXCR2: Prevention of reperfusion injury. Proc. Natl. Acad. Sci. USA.

[122] Ginestier C., Liu S., Diebel M.E., Korkaya H., Luo M., Brown M., Wicinski J., Cabaud O., Charafe-Jauffret E., Birnbaum D. (2010). CXCR1 blockade selectively targets human breast cancer stem cells in vitro and in xenografts. J. Clin. Investig..

[123] Brandolini L., Cristiano L., Fidoamore A., De Pizzol M., Di Giacomo E., Florio T.M., Confalone G., Galante A., Cinque B., Benedetti E. (2015). Targeting CXCR1 on breast cancer stem cells: Signaling pathways and clinical application modelling. Oncotarget.

[124] Schott A.F., Goldstein L.J., Cristofanilli M., Ruffini P.A., McCanna S., Reuben J.M., Perez R.P., Kato G., Wicha M. (2017). Phase Ib Pilot Study to Evaluate Reparixin in Combination with Weekly Paclitaxel in Patients with HER-2-Negative Metastatic Breast Cancer. Clin. Cancer Res..

[125] Bilancia D., Rosati G., Dinota A., Germano D., Romano R., Manzione L. (2007). Lapatinib in breast cancer. Ann. Oncol..

[126] Korkaya H., Wicha M.S. (2013). HER2 and breast cancer stem cells: More than meets the eye. Cancer Res..

[127] Park S.Y., Kim M.J., Park S.A., Kim J.S., Min K.N., Kim D.K., Lim W., Nam J.S., Sheen Y.Y. (2015). Combinatorial TGF-beta attenuation with paclitaxel inhibits the epithelial-to-mesenchymal transition and breast cancer stem-like cells. Oncotarget.

[128] Asiedu M.K., Ingle J.N., Behrens M.D., Radisky D.C., Knutson K.L. (2011). TGFbeta/TNF(alpha)-mediated epithelial-mesenchymal transition generates breast cancer stem cells with a claudin-low phenotype. Cancer Res..

[129] Scheel C., Eaton E.N., Li S.H., Chaffer C.L., Reinhardt F., Kah K.J., Bell G., Guo W., Rubin J., Richardson A.L. (2011). Paracrine and autocrine signals induce and maintain mesenchymal and stem cell states in the breast. Cell.

[130] Akhurst R.J., Hata A. (2012). Targeting the TGFbeta signalling pathway in disease. Nature reviews. Drug Discov..

[131] Anderton M.J., Mellor H.R., Bell A., Sadler C., Pass M., Powell S., Steele S.J., Roberts R.R., Heier A. (2011). Induction of heart valve lesions by small-molecule ALK5 inhibitors. Toxicol. Pathol..

[132] Gueorguieva I., Cleverly A.L., Stauber A., Sada Pillay N., Rodon J.A., Miles C.P., Yingling J.M., Lahn M.M. (2014). Defining a therapeutic window for the novel TGF-beta inhibitor LY2157299 monohydrate based on a pharmacokinetic/pharmacodynamic model. Br. J. Clin. Pharmacol..

[133] Wright C., Moore R.D. (1990). Disulfiram treatment of alcoholism. Am. J. Med..

[134] Han D., Wu G., Chang C., Zhu F., Xiao Y., Li Q., Zhang T., Zhang L. (2015). Disulfiram inhibits TGF-beta-induced epithelial-mesenchymal transition and stem-like features in breast cancer via ERK/NF-kappaB/Snail pathway. Oncotarget.

[135] Zhang D., Sun B., Zhao X., Cui Y., Xu S., Dong X., Zhao J., Meng J., Jia X., Chi J. (2012). Secreted CLU is associated with the initiation of triple-negative breast cancer. Cancer Boil. Ther..

[136] Lenferink A.E., Cantin C., Nantel A., Wang E., Durocher Y., Banville M., Paul-Roc B., Marcil A., Wilson M.R., O’Connor-McCourt M.D. (2010). Transcriptome profiling of a TGF-beta-induced epithelial-to-mesenchymal transition reveals extracellular clusterin as a target for therapeutic antibodies. Oncogene.

[137] Wang C., Jiang K., Kang X., Gao D., Sun C., Li Y., Sun L., Zhang S., Liu X., Wu W. (2012). Tumor-derived secretory clusterin induces epithelial-mesenchymal transition and facilitates hepatocellular carcinoma metastasis. Int. J. Biochem. Cell Boil..

[138] Aalders K.C., Tryfonidis K., Senkus E., Cardoso F. (2017). Anti-angiogenic treatment in breast cancer: Facts, successes, failures and future perspectives. Cancer Treat. Rev..

[139] Conley S.J., Gheordunescu E., Kakarala P., Newman B., Korkaya H., Heath A.N., Clouthier S.G., Wicha M.S. (2012). Antiangiogenic agents increase breast cancer stem cells via the generation of tumor hypoxia. Proc. Natl. Acad. Sci. USA.

[140] Xiang L., Gilkes D.M., Chaturvedi P., Luo W., Hu H., Takano N., Liang H., Semenza G.L. (2014). Ganetespib blocks HIF-1 activity and inhibits tumor growth, vascularization, stem cell maintenance, invasion, and metastasis in orthotopic mouse models of triple-negative breast cancer. J. Mol. Med..

[141] Samanta D., Gilkes D.M., Chaturvedi P., Xiang L., Semenza G.L. (2014). Hypoxia-inducible factors are required for chemotherapy resistance of breast cancer stem cells. Proc. Natl. Acad. Sci. USA.

[142] Zhang C., Samanta D., Lu H., Bullen J.W., Zhang H., Chen I., He X., Semenza G.L. (2016). Hypoxia induces the breast cancer stem cell phenotype by HIF-dependent and ALKBH5-mediated m^6^A-demethylation of NANOG mRNA. Proc. Natl. Acad. Sci. USA.

[143] Nardecchia S., Sanchez-Moreno P., Vicente J., Marchal J.A., Boulaiz H. (2019). Clinical Trials of Thermosensitive Nanomaterials: An Overview. Nanomaterials.

[144] Schmid P., Adams S., Rugo H.S., Schneeweiss A., Barrios C.H., Iwata H., Dieras V., Hegg R., Im S.A., Shaw Wright G. (2018). Atezolizumab and Nab-Paclitaxel in Advanced Triple-Negative Breast Cancer. N. Engl. J. Med..

[145] Zhang Y., Zhang H., Wang X., Wang J., Zhang X., Zhang Q. (2012). The eradication of breast cancer and cancer stem cells using octreotide modified paclitaxel active targeting micelles and salinomycin passive targeting micelles. Biomaterials.

[146] Lee H., Lytton-Jean A.K., Chen Y., Love K.T., Park A.I., Karagiannis E.D., Sehgal A., Querbes W., Zurenko C.S., Jayaraman M. (2012). Molecularly self-assembled nucleic acid nanoparticles for targeted in vivo siRNA delivery. Nat. Nanotechnol..

[147] Muntimadugu E., Kumar R., Saladi S., Rafeeqi T.A., Khan W. (2016). CD44 targeted chemotherapy for co-eradication of breast cancer stem cells and cancer cells using polymeric nanoparticles of salinomycin and paclitaxel. Colloids Surf. B Biointerfaces.

[148] Han N.K., Shin D.H., Kim J.S., Weon K.Y., Jang C.Y., Kim J.S. (2016). Hyaluronan-conjugated liposomes encapsulating gemcitabine for breast cancer stem cells. Int. J. Nanomed..

[149] Ghatak S., Misra S., Toole B.P. (2002). Hyaluronan oligosaccharides inhibit anchorage-independent growth of tumor cells by suppressing the phosphoinositide 3-kinase/Akt cell survival pathway. J. Boil. Chem..

[150] Misra S., Heldin P., Hascall V.C., Karamanos N.K., Skandalis S.S., Markwald R.R., Ghatak S. (2011). Hyaluronan-CD44 interactions as potential targets for cancer therapy. FEBS J..

[151] Bottai G., Truffi M., Corsi F., Santarpia L. (2017). Progress in nonviral gene therapy for breast cancer and what comes next?. Expert Opin. Boil. Ther..

[152] Savas P., Salgado R., Denkert C., Sotiriou C., Darcy P.K., Smyth M.J., Loi S. (2016). Clinical relevance of host immunity in breast cancer: From TILs to the clinic. Nature reviews. Clin. Oncol..

[153] Kobold S., Grassmann S., Chaloupka M., Lampert C., Wenk S., Kraus F., Rapp M., Duwell P., Zeng Y., Schmollinger J.C. (2015). Impact of a New Fusion Receptor on PD-1-Mediated Immunosuppression in Adoptive T Cell Therapy. J. Natl. Cancer Inst..

[154] Iwamura K., Kato T., Miyahara Y., Naota H., Mineno J., Ikeda H., Shiku H. (2012). siRNA-mediated silencing of PD-1 ligands enhances tumor-specific human T-cell effector functions. Gene Ther..

[155] Essand M., Loskog A.S. (2013). Genetically engineered T cells for the treatment of cancer. J. Intern. Med..

[156] Klebanoff C.A., Rosenberg S.A., Restifo N.P. (2016). Prospects for gene-engineered T cell immunotherapy for solid cancers. Nat. Med..

[157] Melero I., Gaudernack G., Gerritsen W., Huber C., Parmiani G., Scholl S., Thatcher N., Wagstaff J., Zielinski C., Faulkner I. (2014). Therapeutic vaccines for cancer: An overview of clinical trials. Nature reviews. Clin. Oncol..

[158] Makkouk A., Weiner G.J. (2015). Cancer immunotherapy and breaking immune tolerance: New approaches to an old challenge. Cancer Res..

[159] Zheng X., Koropatnick J., Chen D., Velenosi T., Ling H., Zhang X., Jiang N., Navarro B., Ichim T.E., Urquhart B. (2013). Silencing IDO in dendritic cells: A novel approach to enhance cancer immunotherapy in a murine breast cancer model. Int. J. Cancer.

[160] Xie Y., Chen Y., Ahmed K.A., Li W., Ahmed S., Sami A., Chibbar R., Tang X., Tao M., Xu J. (2013). Potent CD4+ T-cell epitope P30 enhances HER2/neu-engineered dendritic cell-induced immunity against Tg1-1 breast cancer in transgenic FVBneuN mice by enhanced CD4+ T-cell-stimulated CTL responses. Cancer Gene Ther..

[161] Chiang C.L., Coukos G., Kandalaft L.E. (2015). Whole Tumor Antigen Vaccines: Where Are We?. Vaccines.

[162] Lanzardo S., Conti L., Rooke R., Ruiu R., Accart N., Bolli E., Arigoni M., Macagno M., Barrera G., Pizzimenti S. (2016). Immunotargeting of Antigen xCT Attenuates Stem-like Cell Behavior and Metastatic Progression in Breast Cancer. Cancer Res..

[163] Ridge S.M., Sullivan F.J., Glynn S.A. (2017). Mesenchymal stem cells: Key players in cancer progression. Mol. Cancer.

[164] Pessina A., Cocce V., Pascucci L., Bonomi A., Cavicchini L., Sisto F., Ferrari M., Ciusani E., Crovace A., Falchetti M.L. (2013). Mesenchymal stromal cells primed with Paclitaxel attract and kill leukaemia cells, inhibit angiogenesis and improve survival of leukaemia-bearing mice. Br. J. Haematol..

[165] Pascucci L., Cocce V., Bonomi A., Ami D., Ceccarelli P., Ciusani E., Vigano L., Locatelli A., Sisto F., Doglia S.M. (2014). Paclitaxel is incorporated by mesenchymal stromal cells and released in exosomes that inhibit in vitro tumor growth: A new approach for drug delivery. J. Control. Release.

[166] Bonomi A., Cocce V., Cavicchini L., Sisto F., Dossena M., Balzarini P., Portolani N., Ciusani E., Parati E., Alessandri G. (2013). Adipose tissue-derived stromal cells primed in vitro with paclitaxel acquire anti-tumor activity. Int. J. Immunopathol. Pharmacol..

[167] Bonomi A., Steimberg N., Benetti A., Berenzi A., Alessandri G., Pascucci L., Boniotti J., Cocce V., Sordi V., Pessina A. (2017). Paclitaxel-releasing mesenchymal stromal cells inhibit the growth of multiple myeloma cells in a dynamic 3D culture system. Hematol. Oncol..

[168] Cocce V., Balducci L., Falchetti M.L., Pascucci L., Ciusani E., Brini A.T., Sisto F., Piovani G., Alessandri G., Parati E. (2017). Fluorescent Immortalized Human Adipose Derived Stromal Cells (hASCs-TS/GFP+) for Studying Cell Drug Delivery Mediated by Microvesicles. Anti-Cancer Agents Med. Chem..

[169] Petrella F., Cocce V., Masia C., Milani M., Sale E.O., Alessandri G., Parati E., Sisto F., Pentimalli F., Brini A.T. (2017). Paclitaxel-releasing mesenchymal stromal cells inhibit in vitro proliferation of human mesothelioma cells. Biomed. Pharmacother..

[170] Scioli M.G., Artuso S., D’Angelo C., Porru M., D’Amico F., Bielli A., Gentile P., Cervelli V., Leonetti C., Orlandi A. (2018). Adipose-derived stem cell-mediated paclitaxel delivery inhibits breast cancer growth. PLoS ONE.

[171] Wu J., Liu Y., Tang Y., Wang S., Wang C., Li Y., Su X., Tian J., Tian Y., Pan J. (2016). Synergistic Chemo-Photothermal Therapy of Breast Cancer by Mesenchymal Stem Cell-Encapsulated Yolk-Shell GNR@HPMO-PTX Nanospheres. ACS Appl. Mater. Interfaces.

[172] Tyciakova S., Matuskova M., Bohovic R., Polakova K., Toro L., Skolekova S., Kucerova L. (2015). Genetically engineered mesenchymal stromal cells producing TNFalpha have tumour suppressing effect on human melanoma xenograft. J. Gene Med..

[173] Chakraborty C., Sharma A.R., Sharma G., Doss C.G.P., Lee S.S. (2017). Therapeutic miRNA and siRNA: Moving from Bench to Clinic as Next Generation Medicine. Mol. Ther. Nucleic Acids.

[174] Din F.U., Aman W., Ullah I., Qureshi O.S., Mustapha O., Shafique S., Zeb A. (2017). Effective use of nanocarriers as drug delivery systems for the treatment of selected tumors. Int. J. Nanomed..

[175] Bouchie A. (2013). First microRNA mimic enters clinic. Nat. Biotechnol..

[176] Singh R., Mo Y.Y. (2013). Role of microRNAs in breast cancer. Cancer Biol. Ther..

[177] Han M., Liu M., Wang Y., Chen X., Xu J., Sun Y., Zhao L., Qu H., Fan Y., Wu C. (2012). Antagonism of miR-21 reverses epithelial-mesenchymal transition and cancer stem cell phenotype through AKT/ERK1/2 inactivation by targeting PTEN. PLoS ONE.

[178] Ma L., Reinhardt F., Pan E., Soutschek J., Bhat B., Marcusson E.G., Teruya-Feldstein J., Bell G.W., Weinberg R.A. (2010). Therapeutic silencing of miR-10b inhibits metastasis in a mouse mammary tumor model. Nat. Biotechnol..

[179] Pham P.V., Phan N.L., Nguyen N.T., Truong N.H., Duong T.T., Le D.V., Truong K.D., Phan N.K. (2011). Differentiation of breast cancer stem cells by knockdown of CD44: Promising differentiation therapy. J. Transl. Med..

[180] Cufi S., Vazquez-Martin A., Oliveras-Ferraros C., Martin-Castillo B., Vellon L., Menendez J.A. (2011). Autophagy positively regulates the CD44^+^CD24^−/low^ breast cancer stem-like phenotype. Cell Cycle.

[181] Kumar D., Shankar S., Srivastava R.K. (2013). Rottlerin-induced autophagy leads to the apoptosis in breast cancer stem cells: Molecular mechanisms. Mol. Cancer.

[182] Battula V.L., Shi Y., Evans K.W., Wang R.Y., Spaeth E.L., Jacamo R.O., Guerra R., Sahin A.A., Marini F.C., Hortobagyi G. (2012). Ganglioside GD2 identifies breast cancer stem cells and promotes tumorigenesis. J. Clin. Investig..

[183] Jiang J., Li H., Qaed E., Zhang J., Song Y., Wu R., Bu X., Wang Q., Tang Z. (2018). Salinomycin, as an autophagy modulator—A new avenue to anticancer: A review. J. Exp. Clin. Cancer Res. CR.

[184] Kai M., Kanaya N., Wu S.V., Mendez C., Nguyen D., Luu T., Chen S. (2015). Targeting breast cancer stem cells in triple-negative breast cancer using a combination of LBH589 and salinomycin. Breast Cancer Res. Treat..

[185] Gong C., Yao H., Liu Q., Chen J., Shi J., Su F., Song E. (2010). Markers of tumor-initiating cells predict chemoresistance in breast cancer. PLoS ONE.

[186] Oak P.S., Kopp F., Thakur C., Ellwart J.W., Rapp U.R., Ullrich A., Wagner E., Knyazev P., Roidl A. (2012). Combinatorial treatment of mammospheres with trastuzumab and salinomycin efficiently targets HER2-positive cancer cells and cancer stem cells. Int. J. Cancer.

[187] Salvador M.A., Wicinski J., Cabaud O., Toiron Y., Finetti P., Josselin E., Lelievre H., Kraus-Berthier L., Depil S., Bertucci F. (2013). The histone deacetylase inhibitor abexinostat induces cancer stem cells differentiation in breast cancer with low Xist expression. Clin. Cancer Res..

[188] Croker A.K., Allan A.L. (2012). Inhibition of aldehyde dehydrogenase (ALDH) activity reduces chemotherapy and radiation resistance of stem-like ALDHhiCD44^+^ human breast cancer cells. Breast Cancer Res. Treat..

[189] Bhat-Nakshatri P., Goswami C.P., Badve S., Sledge G.W., Nakshatri H. (2013). Identification of FDA-approved drugs targeting breast cancer stem cells along with biomarkers of sensitivity. Sci. Rep..

[190] Bryan M., Pulte E.D., Toomey K.C., Pliner L., Pavlick A.C., Saunders T., Wieder R. (2011). A pilot phase II trial of all-trans retinoic acid (Vesanoid) and paclitaxel (Taxol) in patients with recurrent or metastatic breast cancer. Investig. New Drugs.

[191] Shimo T., Kurebayashi J., Kanomata N., Yamashita T., Kozuka Y., Moriya T., Sonoo H. (2014). Antitumor and anticancer stem cell activity of a poly ADP-ribose polymerase inhibitor olaparib in breast cancer cells. Breast Cancer.

[192] Ho M.M., Ng A.V., Lam S., Hung J.Y. (2007). Side population in human lung cancer cell lines and tumors is enriched with stem-like cancer cells. Cancer Res..

[193] Matsui W., Wang Q., Barber J.P., Brennan S., Smith B.D., Borrello I., McNiece I., Lin L., Ambinder R.F., Peacock C. (2008). Clonogenic multiple myeloma progenitors, stem cell properties, and drug resistance. Cancer Res..

[194] Saeki T., Nomizu T., Toi M., Ito Y., Noguchi S., Kobayashi T., Asaga T., Minami H., Yamamoto N., Aogi K. (2007). Dofequidar fumarate (MS-209) in combination with cyclophosphamide, doxorubicin, and fluorouracil for patients with advanced or recurrent breast cancer. J. Clin. Oncol..

[195] Katayama R., Koike S., Sato S., Sugimoto Y., Tsuruo T., Fujita N. (2009). Dofequidar fumarate sensitizes cancer stem-like side population cells to chemotherapeutic drugs by inhibiting ABCG2/BCRP-mediated drug export. Cancer Sci..

[196] Gasca J., Flores M.L., Giraldez S., Ruiz-Borrego M., Tortolero M., Romero F., Japon M.A., Saez C. (2016). Loss of FBXW7 and accumulation of MCL1 and PLK1 promote paclitaxel resistance in breast cancer. Oncotarget.

[197] Liu Y., Mallampalli R.K. (2016). Small molecule therapeutics targeting F-box proteins in cancer. Semin. Cancer Boil..

[198] Yin H., Glass J. (2011). The phenotypic radiation resistance of CD44+/CD24(-or low) breast cancer cells is mediated through the enhanced activation of ATM signaling. PLoS ONE.

[199] Li Y., Zhang T., Korkaya H., Liu S., Lee H.F., Newman B., Yu Y., Clouthier S.G., Schwartz S.J., Wicha M.S. (2010). Sulforaphane, a dietary component of broccoli/broccoli sprouts, inhibits breast cancer stem cells. Clin. Cancer Res..

[200] Lan A., Li W., Liu Y., Xiong Z., Zhang X., Zhou S., Palko O., Chen H., Kapita M., Prigge J.R. (2016). Chemoprevention of oxidative stress-associated oral carcinogenesis by sulforaphane depends on NRF2 and the isothiocyanate moiety. Oncotarget.

[201] Guo S., Lu J., Subramanian A., Sonenshein G.E. (2006). Microarray-assisted pathway analysis identifies mitogen-activated protein kinase signaling as a mediator of resistance to the green tea polyphenol epigallocatechin 3-gallate in her-2/neu-overexpressing breast cancer cells. Cancer Res..

[202] Kim J., Zhang X., Rieger-Christ K.M., Summerhayes I.C., Wazer D.E., Paulson K.E., Yee A.S. (2006). Suppression of Wnt signaling by the green tea compound (−)-epigallocatechin 3-gallate (EGCG) in invasive breast cancer cells. Requirement of the transcriptional repressor HBP1. J. Boil. Chem..

[203] Fu Y., Chang H., Peng X., Bai Q., Yi L., Zhou Y., Zhu J., Mi M. (2014). Resveratrol inhibits breast cancer stem-like cells and induces autophagy via suppressing Wnt/beta-catenin signaling pathway. PLoS ONE.

[204] Pandey P.R., Okuda H., Watabe M., Pai S.K., Liu W., Kobayashi A., Xing F., Fukuda K., Hirota S., Sugai T. (2011). Resveratrol suppresses growth of cancer stem-like cells by inhibiting fatty acid synthase. Breast Cancer Res. Treat..

[205] Kakarala M., Brenner D.E., Korkaya H., Cheng C., Tazi K., Ginestier C., Liu S., Dontu G., Wicha M.S. (2010). Targeting breast stem cells with the cancer preventive compounds curcumin and piperine. Breast Cancer Res. Treat..

